# Spontaneous preterm birth: Involvement of multiple feto-maternal tissues and organ systems, differing mechanisms, and pathways

**DOI:** 10.3389/fendo.2022.1015622

**Published:** 2022-10-13

**Authors:** Manuel S. Vidal, Ryan C. V. Lintao, Mary Elise L. Severino, Ourlad Alzeus G. Tantengco, Ramkumar Menon

**Affiliations:** ^1^ Department of Biochemistry and Molecular Biology, College of Medicine, University of the Philippines, Manila, Philippines; ^2^ Division of Basic Science and Translational Research, Department of Obstetrics and Gynecology, University of Texas Medical Branch at Galveston, Galveston, TX, United States

**Keywords:** preterm birth, premature labor, early delivery, pregnancy, parturition, inflammation

## Abstract

Survivors of preterm birth struggle with multitudes of disabilities due to improper *in utero* programming of various tissues and organ systems contributing to adult-onset diseases at a very early stage of their lives. Therefore, the persistent rates of low birth weight (birth weight < 2,500 grams), as well as rates of neonatal and maternal morbidities and mortalities, need to be addressed. Active research throughout the years has provided us with multiple theories regarding the risk factors, initiators, biomarkers, and clinical manifestations of spontaneous preterm birth. Fetal organs, like the placenta and fetal membranes, and maternal tissues and organs, like the decidua, myometrium, and cervix, have all been shown to uniquely respond to specific exogenous or endogenous risk factors. These uniquely contribute to dynamic changes at the molecular and cellular levels to effect preterm labor pathways leading to delivery. Multiple intervention targets in these different tissues and organs have been successfully tested in preclinical trials to reduce the individual impacts on promoting preterm birth. However, these preclinical trial data have not been effectively translated into developing biomarkers of high-risk individuals for an early diagnosis of the disease. This becomes more evident when examining the current global rate of preterm birth, which remains staggeringly high despite years of research. We postulate that studying each tissue and organ in silos, as how the majority of research has been conducted in the past years, is unlikely to address the network interaction between various systems leading to a synchronized activity during either term or preterm labor and delivery. To address current limitations, this review proposes an integrated approach to studying various tissues and organs involved in the maintenance of normal pregnancy, promotion of normal parturition, and more importantly, contributions towards preterm birth. We also stress the need for biological models that allows for concomitant observation and analysis of interactions, rather than focusing on these tissues and organ in silos.

## 1 Introduction

Human parturition is a complex and perplexing physiological phenomenon that involves two independent lives harmoniously residing in one body for ~40 weeks. This harmonious state between the mother and her fetus is established by endocrine mediators, adaptive and innate immune system components, complement and vascular biological processes, extracellular matrix and matrix remodelers, factors involved in cell growth, differentiation, transition, migration, and invasion, redox and electrophysiologic balance, and various exocrine and paracrine signalers (1–4). The maternal systems support fetal growth and development during this period with a steady supply of all essential and indispensable compounds, which are selected and supplied to the fetus without hindrance. Multitudes of physiologic processes generate a well-balanced and regulated inflammation, primarily localized to specific feto-maternal tissue and organ systems function and involves changes in oxygen tension generating reactive oxygen species radicals. A combination of inflammation and oxidative stress caused by reactive oxygen species is necessary for feto-placental growth and pregnancy maintenance (5). Maternal tissue and organ systems, such as the cervix, myometrium, and decidua, and fetal organ systems, such as the fetal membranes (amniochorion) and placenta, synchronously contribute to pregnancy wellbeing and ensure protection from all exogenous and endogenous factors. The gestational period is a ‘lifetime’ for fetal organs where they grow, develop, and get delivered along with the fetus. On the contrary, maternal tissues and organ systems repair, remodel and revert to a non-pregnant state for future reproductive purposes. This suggests that the fate of these tissues and organs is vastly different and their physiologic trajectory for ending their roles leading to parturition is also different. Towards term, both feto-maternal tissue and organ systems tend to transition from their quiescent status that maintains pregnancy to active status to facilitate labor and delivery. However, this transition is not the same in each feto-maternal tissue (6–8). Inflammation is the underlying mechanism to transition from a quiescent state to an active state or to remain active once that phase is achieved (5). The initiators, activators, and effectors contributing to the inflammatory events transitioning them to a pro-parturition phenotype are different in each feto-maternal system.

Over the decades, multitudes of factors have been reported to contribute to transition processes in each feto-maternal system. All these studies aimed to understand pregnancies that end preterm (prior to 37 weeks of gestation). The rationale is that understanding normal term labor and delivery (term parturition) may lead to an understanding of preterm birth (PTB). The latter is a complex syndrome initiated by either known (indicated) or unknown factors (spontaneous) (2). Studies are needed to understand PTB due to the high rate of its incidence (~12% globally annually), mortality and morbidity to mothers and neonates, survivors having physical and intellectual handicaps, and issues related to fetal programming contributing to adult-onset diseases (9). Spontaneous onset of labor leading to PTB is the most difficult to predict due to heterogeneities associated with various factors that include, but are not limited to, etiologies, genetic and environmental interactions, mechanistic pathophysiologies, biomarkers, and clinical presentations (2, 10). Even with advancements in clinical management, research, and development in perinatal medicine and research, spontaneous PTB (sPTB) remains a major clinical dilemma.

Knowledge gained on the mechanism of normal term parturition has not been translatable easily to sPTB mechanisms. This suggests that the initiators of events culminating in sPTB are not the same as reported in term parturition, although overlap may be observed between the two phenotypes in several areas. sPTB has two states of risk factors that the mother experience. One is static, comprising risk factors that are generally stable and unlikely to change during pregnancy. These include maternal race, genetics, geographic location, environment, socio-economic factors, behavioral factors, nutrition, and obesity (10, 11). The other is dynamic, which includes infection, stress, hydramnios, allergies, rupture of the membranes, and bleeding, to name a few. The interaction between the static risk factors and pregnancy environment or exposure to infection-like conditions during pregnancy can manifest into a pathophysiologic mechanism contributing to a dynamic risk state (10, 11). The effector pathways of these interactions resulting in labor and delivery preterm are the same. So, the question remains – what really results is different in sPTB, and why do we have great difficulty in understanding sPTB pathology? It is true that many disease processes contributing to sPTB syndrome may be initiated in one or a few tissue and organ systems or called the ‘disease of that specific system,’ or even more broadly ‘disease of the mother,’ or ‘disease of the fetus.’ Investigators in the field of perinatal research have invested in studying these systems in silos and contributed a tremendous wealth of data to our understanding. Despite the fact that the previous designations are correct, these can also contribute to ambiguity – biomarkers and interventions are siloed to a specific system without considering the involvement of other systems as well as intersystem interactions, both of which are essential to maintaining pregnancy. The collapse of homeostasis in each system is required to promote labor at term and preterm. Thus, this review provides an in-depth analysis of various disease processes in each fetal and maternal tissue and organ system and how these may manifest in the PTB pathway.

## 2 Preterm birth is a disease of the fetal organs

### 2.1 Preterm birth: A disease of the placenta

#### 2.1.1 Structure and function of the placenta during pregnancy

The placenta has been an organ of interest due to its unique nature of spontaneous formation during pregnancy. This fetally derived organ develops during the early implantation period as a mass of inner cytotrophoblasts (CTs) and outer syncytiotrophoblast (SCT) layer. The SCT forms a massive sheet of primary syncytium that slowly advances towards the decidua and gradually invades the tissue space and the innervating blood vessels to create early maternal-fetal hematological connection called lacunae (12). A subpopulation of CTs begins projecting outward to form CT columns called placental villi, with their core allowing for penetration of fetal capillaries. Distal villous CTs differentiate into extravillous trophoblasts (EVTs), allowing for further invasion into the maternal decidua. The CTs also project laterally, creating an outer CT shell (13). Eventually, the basal side of the CT shell regresses, and the apical portion remains as an attached plate to the decidua (14).

Primarily, the establishment of feto-maternal circulation in the intervillous spaces is at the heart of the function of the placenta (15). Maternal blood bathes these spaces, where gas exchange, nutrient transport, and waste disposal occur secondary to SCT, and to a lesser extent, to CT functions (14). The SCT also secretes a milieu of canonical steroids (e.g., estrogen, progesterone), neurosteroids (e.g., allopregnanolone), peptides (e.g., corticotropin-releasing hormone (CRH), oxytocin), and placenta-specific hormones (e.g., human chorionic gonadotropin (hCG), human placental lactogen (hPL), to name a few (16, 17). The SCT also provide a physical barrier against microbes that may be carried through the maternal blood (18). Thus, the placenta mediates multiple functions critical to the survival of the growing fetus.

#### 2.1.2 Placental contributions to parturition

Changes in various hormonal cascades become more critical in the placenta as parturition draws near; in particular, two placental hormones are deemed necessary in the bringing forth the fetus. The first hormone, CRH, appears to be the master regulator of birth timing. Its production is under control by cortisol, a glucocorticoid hormone typically denoted as a stress signal (19, 20). Unlike maternal CRH, which is negatively regulated by maternal cortisol, placental CRH is positively regulated by cortisol, providing a feed-forward mechanism that does not have an “off-switch” as the pregnancy nears term (21). Placental CRH is locally transported to other feto-maternal compartments and exerts its local actions through CRH receptors distributed along with those tissues (22–24). In the myometrium, CRH enhances the production of cytokines and chemokines that promote inflammation and increase the output of contractility-associated proteins; however, multiple studies are contrasting in terms of how CRH mediates contractility and relaxation in the term and preterm myometrium (25–28). In the decidua, early CRH induces decidualization and implantation *via* interleukin IL-6 and IL-1β stimulation, while near term, CRH promotes prostaglandin E2 (PGE2) production contributing to birth (29–31). In the fetal membranes, placental CRH actions on various receptor subtypes found in the amnion epithelium may lead to the production of PGE2 from the membranes secondary to cyclooxygenase-2 (COX-2) activation (29, 30, 32). Additionally, CRH also induces the expression of prostaglandin H synthase 2 (PGHS2), which contributes to local prostaglandin output (33).

Progesterone, the essential hormone for the maintenance of pregnancy, downregulates placental CRH production without affecting prostaglandin dehydrogenase (PGDH) expression that contributes to pregnancy maintenance (34, 35). Progesterone exerts actions toward its cognate placental receptors PR-A and PR-C; PR-B expression, on the other hand, appears to be tonically repressed in human placental cells (36–38). PR-A inhibits progesterone action in the placenta, directing the activation of non-canonical NF-κB signaling *via* NF-κB inducing kinase (NIK)/RelB/NF-kB2 and subsequently leading to COX-2 activation (39–42). Fetal exosomes containing platelet-activating factor (PAF) have been shown to decrease PR-A expression in term and preterm placentas epigenetically (42). As placental progesterone appears only to diminish after placental expulsion, it can be surmised that functional progesterone withdrawal in the placenta, as pertained to by the decrease in PR-A: PR-B ratio, may also contribute to parturition (40). However, the functional progesterone theory still remains controversial due to its unclear mechanisms; for instance, in other tissues such as the myometrium, PR-A is observed to promote inflammatory cascades in contrast to mechanisms in the placenta (43, 44). Besides, CTs contain progesterone receptor membrane components (PGRMCs), which play a major role in progesterone function in a cell. The role of these receptors has not been fully investigated yet to elucidate functional progesterone withdrawal. The inhibition of progesterone action in the placenta, regardless of the mechanism, allows for the accumulation of placental CRH (“placental aging”) and may aid in the progression of labor.

#### 2.1.3 Disease states in the placenta contributing to sPTB

##### 2.1.3.1 Placental inflammation

Bacteria can hematogenously access the placental intervillous spaces from the maternal blood flow. However, the syncytiotrophoblast that outlines the placental villi provide a physical barrier against infection (18). Additionally, the branched arrangement of the microvilli, absence of intercellular junctions, robust physical actin network, and autophagy initiation may provide additional protection (45, 46). However, some bacteria may cross the placenta by hijacking the autophagy system or by infecting EVTs on the placental-decidual interface, which offers less protection compared to syncytiotrophoblasts (47–50).

It is important to note that even in the absence of a microbial etiology, sterile inflammation may still occur that can trigger sPTB (51, 52). For instance, chronic villitis has been described as pathologic findings in some cases of sPTB, and it was initially highlighted that a non-infectious etiology can bring about this pathology (53). Chronic villitis with unknown etiology has been described as an inflammation secondary to abnormal upregulation of mediators, resulting to infiltration of macrophages and T cells (54). It has been suggested that this is an allograft reaction, not necessarily an epitope-specific response similar to an immune cell response to viral or bacterial epitopes (55).

Regardless of infectious or non-infectious etiology, placental inflammatory responses may lead to immune rejection and failure of placental adaptation (56, 57). Damage-associated molecular patterns (DAMPs), or alarmins, are crucial in the inflammatory response leading to sPTB. Stress signaler p38 mitogen-activated protein kinase (MAPK) and high-mobility group box 1 (HMGB1) proteins enhance inflammatory responses in the placenta *via* cytokine and uterotonin production (58–63). Cytokine expression in sPTB is centered around the critical NF-kB pathway (64). This ubiquitous pathway inhibits the activity of 11β-hydroxysteroid dehydrogenase 2 (11β-HSD2), the enzyme responsible for converting cortisol to inactive cortisone (65, 66). Therefore, inflammation provides a feed-forward mechanism that allows cortisol and CRH to accumulate in the placenta (67–69). In the canonical setting, NF-κB also positively regulates the production of pro-inflammatory cytokines tumor necrosis factor-alpha (TNF-α) and IL-6 from trophoblasts (70–72). IL-8 appears to be upregulated in preterm placenta extracellular vesicles, indicating chemotactic functions to allow inflammatory infiltration in the placenta (73). IL-10, an anti-inflammatory cytokine, is observed to be decreased in human placental explants in PTB; its actions are thought to occur *via* selective nuclear translocation of an NF-κB homodimer repressor (74, 75). The importance of IL-10 is highlighted in IL-10-knockout mouse models, which appear to be more susceptible to lipopolysaccharide-induced sPTB (76). An interplay of dysregulations in cytokine expression in the placenta may promote sPTB.

Due to inflammatory activation in the placenta, downstream effectors of cervical and fetal membrane remodeling may also be induced by inflammatory mediators produced from the placenta. Placental production of matrix metalloproteinases (MMPs) and prostaglandins may be superimposed with the production of these molecules in other feto-maternal compartments during PTB (69, 77, 78). In the preterm placenta, protein and gene expression of MMP-1 and MMP-2 were significantly higher than in the term placenta (79). TNF-α and IL-6 may also direct the production of PGE2 and the reduction of PGDH in trophoblasts (71, 72, 80). IL-10 reduction also coincides with a decrease in PGE2 expression in trophoblasts (74, 75). Increases in PGF2α and PGE2 have been documented in the preterm setting, thought to occur *via* an increase in phosphatidylinositol-3 kinase (PI3K)/protein kinase B (Akt) signaling; a placental increase in prostaglandin concentration may contribute to decidual senescence and further promotes a pro-inflammatory environment (77, 81).

##### 2.1.3.2 Placental endocrine dysfunction

Multiple evidence points out a potential involvement of endocrine dysregulation in sPTB patients. For instance, CRH elevation has been consistently implicated as a contributor to sPTB. Patients who had sPTBs have an almost sixfold increase in CRH concentrations, observed at 28-33 gestational weeks, compared to those in term labor (82–85). In patients with recurrent preterm birth, elevated CRH levels were demonstrated to have an almost similar area under the receiver operating characteristic curve compared to a previous risk of preterm birth (86). Accelerated placental CRH upregulation may lead to an earlier initiation of myometrial contractility and cervical ripening that contributes to sPTB (87, 88). Maternal stress has been pointed out as a possible contributor to abrupt increases in placental CRH, although the evidence remains conflicted (89). Therefore, mechanisms of endocrine dysfunction remain to be elucidated.

Some studies have shown alterations in the expression of progesterone receptors and associated receptors in preterm placentas. (Papamitsou et al., 2011). PR-A expression is also observed to decrease in relation to PR-B; however, the low expression of PR-B posits a question as to the robustness of monitoring changes in these isoforms in the context of preterm birth (90). Instead, there is growing evidence to support the associated receptor progesterone receptor membrane component 1 (PGRMC1) (90). Polymorphisms in these receptors may explain their involvement, but other factors must be considered, including fetal sex and ethnicity (91, 92). Nonetheless, the proposed involvement of progesterone receptor isoforms and subtypes in PTB remains up for debate.

##### 2.1.3.3 Uteroplacental insufficiency

Uteroplacental insufficiency may also contribute to preterm labor, such as those occurring in patients with placental implantation abnormalities or Müllerian anomalies that manifest as lesions of malperfusions in the placenta (93, 94). Patients with placental implantation abnormalities, such as shallow invasion in the decidua or placenta previa, have been shown to have higher rates of preterm delivery and serve as an independent risk factor for subsequent sPTB (95, 96). Patients with Müllerian anomalies may lead to aberrant implantation due to decreased uterine muscle mass that somehow poorly controls placental invasion (94, 97). In this setting of blood flow insufficiency, the renin-angiotensin pathway may play a role in the pathophysiology leading to sPTB. The vascular tone of uteroplacental arteries is under the control of this pathway, and increased activity of this in the decidua may induce vasoconstriction and eventual blood flow insufficiency in the placental unit (98, 99) with subsequent increase in vascular reactivity, mediated by the renin-angiotensin signaling pathway and PI3K/Akt/phosphatase and tensin homolog deleted on chromosome 10 (PTEN) pathway. This pathway has been extensively discussed in the context of preeclampsia, with PTEN upregulation *via* the NF-κB pathway inhibiting proper trophoblast invasion and Akt downregulation leading to endothelial dysfunction; as this is another pathway involved in angiogenesis apart from the canonical vascular endothelial growth factor (VEGF), PI3K/Akt/PTEN pathway may play a role *in utero*placental insufficiency in preterm patients (100–102). Moreover, placental endothelial cells also displayed a reduction in VEGF staining with a concomitant increase in receptor tyrosine kinase with immunoglobulin and epidermal growth factor homology domains-2 (TIE-2) staining in placentas with PTB (103). Placental insufficiency may serve as a stress signal that increases CRH levels, as seen in preeclamptic patients; and may lead to CRH-specific downstream effects; however, as the placenta adapts by altering its vasculature to accommodate under perfused areas, it remains to be seen whether a histological diagnosis of insufficiency is a risk factor for sPTB itself (93, 101, 104).

#### 2.1.4 Summary

**Figure 1:**
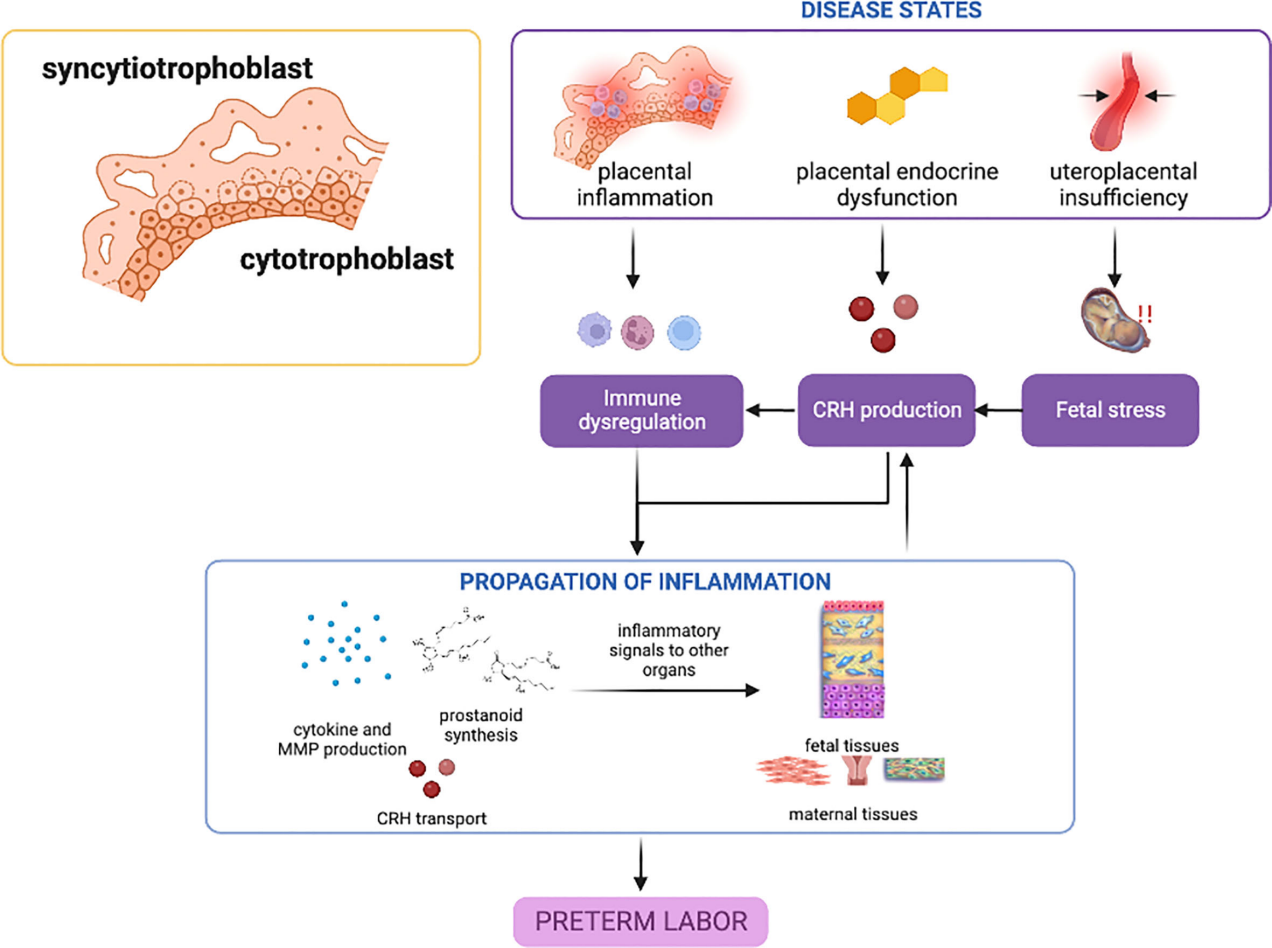
Disease states in the placenta contributing to preterm birth. Placental inflammation, endocrine dysfunction, and uteroplacental insufficiency can lead to inflammatory activation and propagation of inflammatory mediators towards other gestational tissues. CRH may play the predominant role in these placental disease states, with inflammation providing a feed-forward mechanism. This figure was created with BioRender.com.

Overall, dysregulations in CRH and progesterone expression and receptor action may be critical players in the role of the placenta in sPTB; mechanisms leading to these phenomena are still unclear, and genetic and epigenetic studies and their interaction with various risk environments or other endogenous environments may shed light on susceptibility in the general population. Nonetheless, placental inflammatory response involving mainly the NF-κB pathway appears to be the primary disease state contributing to sPTB. (**Figure 1**).

### 2.2 Preterm birth: A disease of the fetal membranes

#### 2.2.1 Fetal membrane structure and function

The fetal, or amniochorionic, membranes are often considered mere appendages of the placenta or dead tissues at delivery (105). However, recent investigations on fetal membranes show that they are highly specialized and essential for protecting the fetus, maintaining pregnancy, and signaling the initiation of parturition (106–108) The fetal membranes are composed of the amnion, a single cell epithelial layer that forms the innermost layer of the intrauterine cavity, and the chorion, a trophoblast layer that includes the feto-maternal interface barrier connected to the maternal decidua. The two layers are connected by a collagen-rich extracellular matrix (ECM) containing amnion and chorion mesenchymal cells (109, 110).

The membrane performs vital mechanical, biochemical, immunological, and endocrine functions throughout gestation—the amnion layer functions as structural and mechanical support for the fetus. Layers of the collagen matrix and cells form a protective barrier and a water-tight seal around the fetus and amniotic fluid (111). Stromal mesenchymal cells form the fibroblast layer and secrete collagen types I, III, and V, providing a fibrous skeletal framework and reinforcing amnion integrity (112). Along with viscoelastic elastin and microfibrils, the membranes withstand stretch from the gradually increasing hydrostatic pressure from the amniotic fluid (113) sudden impacts, strains, and compressions from fetal movement. Additionally, the amnion might also be involved in amniotic fluid homeostasis by converting bicarbonate to CO_2_ with human carbonic anhydrase isoenzymes, abundantly expressed in amnion epithelial cells (AECs), allowing for pH regulation and fluid turnover (9). Furthermore, the membranes protect the fetus from environmental or endogenous sources of physical, chemical, and biological hazards (114). The amniochorion protects the fetus from pathogens in several ways, one being structurally impermeable to pathogens, and by expression of antimicrobial peptides (AMPs) that target microorganisms in the membrane or amniotic fluid.

As a supplementary endocrine organ, the fetal membranes are rich sources of prostaglandin and cortisol (115, 116). The amnion epithelium is a significant site of prostaglandin synthesis and metabolism (117, 118). The amount of prostaglandins that reach the myometrium is dependent on the expression and activity of (1) PGHS in the amnion, and to a lesser extent, in the chorion and decidua for prostaglandin biosynthesis; and (2) PGDH in chorion trophoblast for prostaglandin inactivation (119–121). Fetal contributions to the overall prostaglandin pool confirm the importance of fetal membranes in parturition as a source of mediators for remodeling the ECM causing fetal membrane weakening and eventual rupture, particularly in the area overlying the cervix (122–125). Moreover, the membranes also appear to have the highest capacity to convert biologically inactive cortisol into active cortisol in late gestation among fetal tissues (126, 127). Fetal membranes express 11β-HSD1 abundantly, and its expression is under feed-forward induction by cortisol potentiated by pro-inflammatory cytokines (128, 129). Expression of 11β-HSD1 in fetal membranes increases with gestational age, and cortisol is further increased during parturition (130, 131). Altogether, fetal contributions to cortisol and prostaglandin pool are significant factors for the progression of labor (132).

#### 2.2.2 Fetal membrane mechanisms in parturition

As the fetal membrane cells grow and multiply, they undergo telomere-dependent cellular senescence resulting in an aging phenotype with concomitant changes in biochemical, molecular, and morphological signatures. Increased oxidative stress in term membranes leads to an irreversible cell fate (senescence) and generate senescence-associated secretory phenotypes (SASPs). SASP comprises of a unique set of biochemicals constituting sterile inflammation leading to membrane deterioration and rupture as part of the final preparatory stages for eventual delivery (133–135). *In vitro* and animal models of amnion epithelial cells have shown that oxidative stress accelerates senescence and the development of SASPs (108, 136). Senescent fetal membranes also release damage-associated molecular patterns (DAMPs), cellular markers that represent cellular injury and tissue damage considered as endogenous danger signals that induce potent inflammatory responses during sterile inflammation (137). DAMPs such as HMGB1, heat shock protein-70 (HSP-70), histone H3, IL-33, and cell-free fetal telomere fragments (cffTFs) are released in senescent amnion cells (135, 138).

Both SASPs and DAMPs are paracrine signalers from the fetal membranes which propagate inflammatory signals to other feto-maternal compartments to promote parturition (105). These signals are effectively propagated *via* extracellular vesicles, specifically by exosomes and microvesicles (139–141). Amnion cells were previously shown to produce exosomes and microvesicles with distinct inflammatory cargo (142). AEC-derived exosomes and microvesicles elicit inflammatory reactions in maternal tissues while having distinct cargo and surface protein profiles (143, 144). Senescent amnion cells exposed to oxidative stress conditions produce contain HMGB1 and cffTFs (145). Aside from local feto-maternal routing, as established by exosome trafficking in animal models (146, 147), systemic routing is also possible, as established by exosome presence in maternal blood (146, 147).

##### 2.2.2.1 Microfracture development and failure of membrane remodeling

The fetal membranes are also sites of microfractures formation. Microfractures are channels or tunnels created by cellular shedding or puckering with degradation of the basement membrane and matrix collagen as a result of the remodeling of membrane cells (148, 149). These microfractures sites allow for cellular recycling and membrane remodeling *via* localized inflammation (148). Epithelial to mesenchymal transitions (EMT) facilitate cellular migration, while mesenchymal-to-epithelial transition (MET) allows remodeling of microfractures and cellular gaps in the amnion layer *via* PGRMC2 and c-Myc mediation (150). However, inflammation and oxidative stress counter-regulate this mechanism and promotes EMT secondary to p38 MAPK activation to increase microfractures along the membranes (151–154). These events also reduce PGRMC2 expression preventing MET, leading to accumulation of mesenchymal cells in the ECM that are highly sensitive to oxidative stress and inflammation.

#### 2.2.3 Preterm birth as a disease of the fetal membranes

##### 2.2.3.1 Premature senescence as a mechanism of membrane-associated sPTB

Senescence and sterile inflammation are physiologic processes in parturition. However, premature activation of inflammatory and oxidative stress pathways is implicated in the pathogenesis of sPTB and prelabor rupture of membranes, a condition associated with 40% of all sPTB. Premature activation increases oxidative stress and accelerates senescence causing SASP/DAMP-associated inflammation in fetal membranes. This has been proven by *in vitro* models exposed to cigarette smoke, an oxidative stress inducer (133, 155, 156), environmental pollutants (134, 151), non-infectious (i.e., sterile) inflammation, and infection (157). Overwhelming redox imbalance from risk factors compromises the ability of the membranes to detoxify reactive oxygen species and repair oxidative damage from these highly reactive molecules (158), leading to telomere shortening (159), p38 MAPK activation, senescence, and inflammation (155, 160). Premature aging propagates inflammatory signals to other fetomaternal tissues and increases the overall inflammatory load, while also promoting membrane dysfunction as demonstrated by increased microfractures that lead to rupture of membranes (148).

##### 2.2.3.2 Chorioamnionitis

Chorioamnionitis, also known as intrauterine infection, is the most common cause of disturbance in the balance of inflammatory processes in the membrane. Chorioamnionitis is a major cause of PTB and commonly results from an ascending infection from the lower genital tract reaching the intrauterine cavity with concomitant infiltration and activation of immune cells (161–166). Neutrophils usually are the predominant infiltrating population in the amnion and choriodecidua (163, 167) which is likely due to increased neutrophilic chemotactic factors such as IL-8 in the amnion (168). These neutrophils release several inflammatory mediators such as IL-8, TNF-α, and macrophage inflammatory protein (MIP)-1β/chemokine (C-C motif) ligand 4 (CCL4) (169, 170) and MMPs (171–175). In combination with a compromise in antimicrobial properties, immune function, inflammatory homeostasis, and membrane structural integrity are detrimental to the pregnancy as it allows for microbial invasion from the genital tract (176) and activation of host inflammatory response leading to collagenolysis-mediated mechanical disruption (173, 177, 178). The resulting membrane weakening predisposes the membranes to rupture (107, 179). More important is that anti-apoptotic signals towards these neutrophils are upregulated, providing a feed-forward mechanism that allows them to persist longer and continue inflammatory signaling (168, 170). Other innate immune cells are also recruited and play a role in the rupture of membranes as macrophages release several MMPs (180–182) and mast cells secrete several modulators (183–185). Alteration in these immunological signatures or function may lead to activation of pro-inflammatory pathways and disruption of immune tolerance which can induce preterm labor (186–188).

##### 2.2.3.3 Fetal membrane pathology secondary to uterine overdistension

Conditions that lead to overdistension of fetal membranes include polyhydramnios and multi-fetal pregnancies. Several lines of evidence suggest a role for overdistension in sPTB. Polyhydramnios or the excessive accumulation of amniotic fluid in pregnancy, and multi-fetal pregnancies are associated with an increased risk of preterm labor and delivery (189–191). Static stretch induces p38 MAPK activation but does not induce senescence or MMP-9 activation which suggests that static stretch contributes to fetal membrane remodeling and growth but not to labor-associated changes (192). Biomechanical studies of human fetal membranes show that stretch induces IL-8 and collagenase activity (193). Overdistension of fetal membranes results in increased production of cyclooxygenase-2 and prostaglandins (194, 195) and induction of terminal pathways in parturition (196, 197). Preterm membranes have a higher sensitivity to external forces and have higher mechanical property heterogeneity compared to term membranes (198). The biomechanical properties of membranes may be a potential factor for increased microfracture formation and premature aging in preterm membranes. Thus, physical stressors such as mechanical stretch also impact membrane function and have a significant contribution to membrane weakening.

#### 2.2.4 Summary

**Figure 2:**
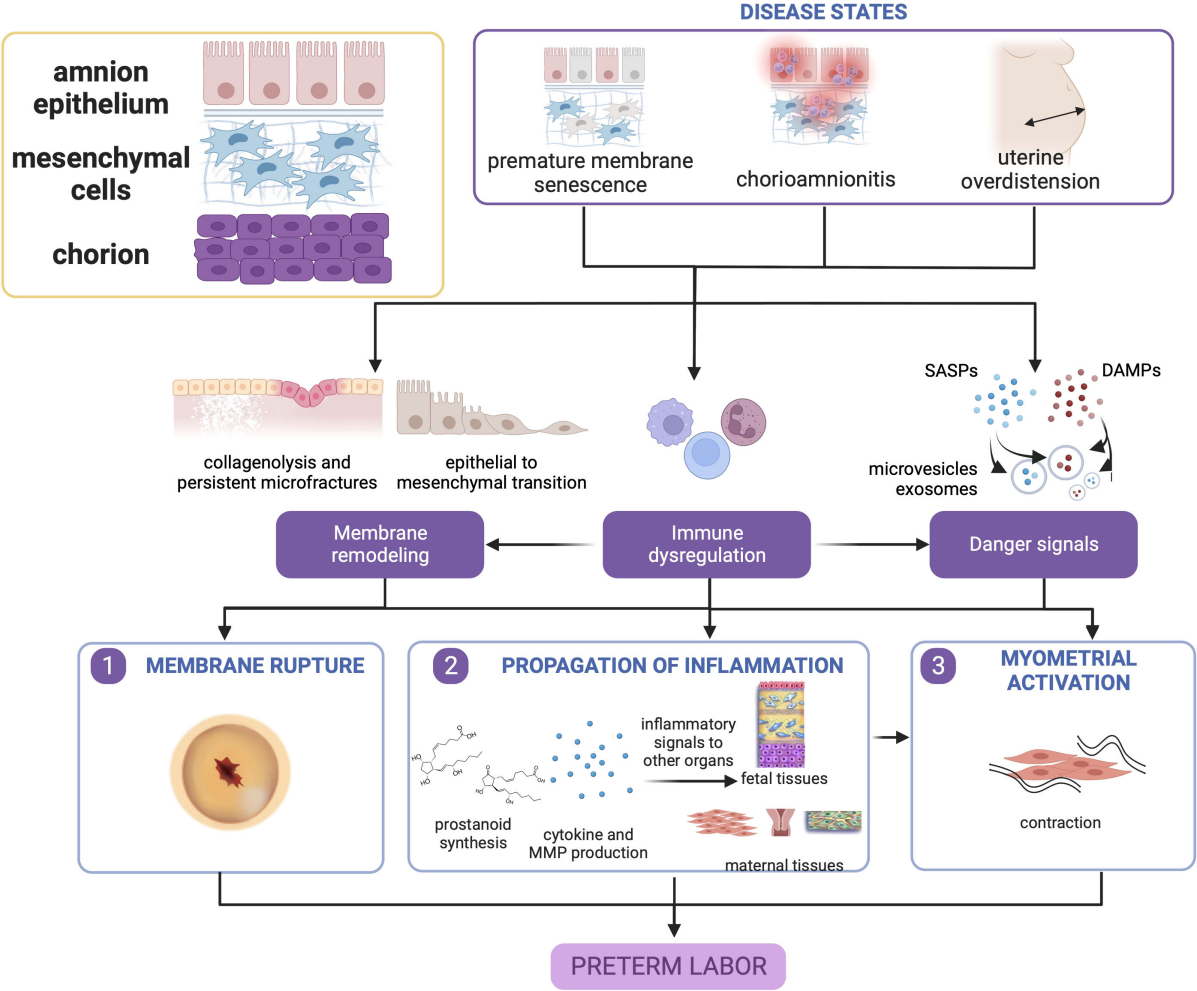
Disease states in the fetal membranes contribute to preterm birth. Premature membrane senescence, chorioamnionitis, and uterine overdistension can all lead to dysfunctional membrane remodeling, generation of danger signals (senescence-associated secretory phenotype [SASP], and damage associate molecular pattern markers [DAMPs], and chemotaxis and immune cell activation. The localized presence of danger signals provides greater contributions toward untimely membrane rupture, while immune cell activation in the presence of danger signals may cause myometrial activation. This figure was created with BioRender.com.

The pathophysiology of these diseases centers around premature activation of pro-inflammatory pathways, disruption of immunologic function, and dysfunction in fetal membrane remodeling. Fetal membrane senescence, and inflammatory and oxidative stress pathways are pivotal to early rupture which inevitably leads to sPTB. Paracrine signals comprised of endocrine and inflammatory mediators are propagated to other feto-maternal compartments to promote parturition. Therefore, disease and dysfunction of the fetal membranes are paramount in the pathophysiology of sPTB as shown in **Figure 2**.

## 3 Preterm birth as a disease of the maternal tissues and organs

### 3.1 Preterm birth: A disease of the decidua

#### 3.1.1 Structure and function of the decidua during pregnancy

The decidua is a modified mucosal lining of the endometrium comprised of terminally differentiated endometrial stromal cells, newly generated maternal vascular cells, and maternal immune cells (199). Decidualization occurs after ovulation during the secretory phase of the menstrual cycle due to increased progesterone levels. Decidualization triggers an influx of decidual leukocytes, mainly specialized uterine natural killer (uNK cells) and dendritic cells, facilitating immunosuppression to prevent fetus rejection and acting as regulators of spiral artery remodeling and endometrial stromal cell differentiation (200). During endometrial stromal cell (ESC) differentiation, the cells undergo phenotypic and functional changes, altering appearance from fibroblast-like to epithelial cell-like due to accumulation of glycogen and lipid droplets, and producing cytokines, growth factors, molecules like insulin-like growth factor-binding protein 1 (IGFBP1) and prolactin, and ECM proteins such as fibronectin and laminin (201). During pregnancy, the decidua facilitates nutrient transport to the placenta, protects the embryo from the immunological responses of the mother, and regulates trophoblast invasion.

#### 3.1.2 Decidual contributions to parturition

During normal labor, the decidua undergoes a series of anatomical and biochemical events in a process called “decidual activation,” where there is an increase in prostanoids, mainly PGF2α and PGE2, proinflammatory cytokines and chemokines; increase in MMP activity leading to ECM protein degradation; and apoptosis (2). Failure to suppress prostaglandin synthesis in the decidua has been associated with adverse pregnancy outcomes (202). In mice, increased synthesis of prostanoids is sufficient to trigger PTB in the absence of progesterone withdrawal. One of the mechanisms for this phenotype in mice is uterine-specific p53 deletion, which activates PI3K/Akt signaling and induces premature decidual senescence *via* mammalian target of rapamycin complex 1 (mTORC1), leading to increased expression of PGF2α *via* activation of COX-2/PGF synthase/PGF2α pathway (81, 203). Another mechanism is a partial loss of function in the gene encoding for 15-hydroxyprostaglandin dehydrogenase (15-HPGD), as evidenced in a hypomorphic mouse model for 15-HPGD, leading to prematurely increased levels of PGF2α and PGE2 (204). Decidual activation eventually weakens the decidual-placental interface, leading to the physical separation of the decidua from the placenta and the fetal membranes (2, 205). Decidual infiltration by immune cells in addition to activation of resident immune cells precedes myometrial infiltration, suggesting that decidual activation facilitates inflammatory events in the myometrium which eventually leads to contractions (206). PGF2α produced by decidual cells upregulate the expression of contraction-associated proteins (CAPs) such as ion channels that increase calcium influx, oxytocin, and prostaglandin receptors, and gap junction proteins such as connexin 43 (Cx43), thus promoting myometrial contractility (207–209). Although mainly produced by the fetal membrane, PGE2 produced by the decidua contributes to cervical ripening due to the proximity of tissues and interaction between cells (210). Apoptosis in the amnion membrane causes an increase in prostaglandin levels, leading to MMP-promoted apoptosis; this results in a positive feedback loop that further drives decidual activation (211). Indeed, it has been posited that the decidua has a role in determining the length of gestation–the so-called “decidual clock” (208).

#### 3.1.3 Disease states of the decidua contributing to sPTB

##### 3.1.3.1 Deciduaitis

Infection and inflammation have been identified as a significant risk factor for sPTB, with clinical and/or histologic evidence of chorioamnionitis seen in 35% of women with early sPTB (28-34 weeks) and 47% of women with very early sPTB (20-28 weeks) (212). In response to infection, the decidual blood vessels have been demonstrated to serve as first responders *via* Toll-like receptor-4 (TLR4) sensing (213). Endothelial-specific deletion of *Tlr4* results in amelioration of preterm birth rates in lipopolysaccharide (LPS)-treated mouse models (213). Resident immune cells in the decidua, relegated as secondary responders instead of being primary targets during infection, become activated upon exposure to pro-inflammatory cytokines (168, 170, 214, 215) and enhance p38 MAPK-mediated MMP release (deciduaitis) (216).

Deciduaitis is one of the conditions associated with sPTB. The established inflammation provides a feed-forward mechanism that allows for intense decidual neutrophil infiltration (217) and monocyte/macrophage infiltration. Inhibition of NF-κB and p38 MAPK attenuates this response (218). IL-1β also inhibits decidual expression of nuclear PRs and enhances decidual expression of COX-2, PGE2, and PGF2α, and in combination with MMP release, may be a summative mechanism in chorioamnionitis-associated PTB (219).

##### 3.1.3.2 Premature decidual senescence

Still, many cases of sPTB occur without placental abruption or infection, leading to alternative hypotheses as to how the decidua may mediate a sterile inflammatory process. Premature decidual senescence as a mechanism for sPTB has been seen in humans, wherein decidua basalis collected from the placenta of women following vaginal delivery at preterm showed increased expression of senescence markers (e.g., senescence-associated beta-galactosidase and phosphorylated histone protein γH2AX) as well as increased expression of COX-2 and phosphorylated ribosomal protein S6, a marker for mTORC1 signaling (220). In a study involving whole-genome microarray analyses on decidual tissues, multiple differentially expressed genes were found among term, preterm in labor, and preterm not in labor tissues (221) Among the top enriched pathways in preterm labor samples were “complement and coagulation cascades” and “cytokine-cytokine receptor interaction,” highlighting the role of inflammatory signaling in preterm labor. Genome-wide association studies (GWAS) showed three loci in maternal GWAS (*EBF1, EEFSEC, AFTR2*) and zero loci in infant GWAS associated with PTB (222–224)Integration of results from GWAS of gestational duration with transcriptome, epigenome, open chromatic, and chromatic interaction annotations of cultured decidual cells uncovered a novel causal locus *HAND2* (Heart And Neural Crest Derivatives Expressed 2) and refined annotation of a previously identified locus that suggests *GATA2* as a likely gene target (224). Despite no statistical differences in immune cell proportions in term and preterm laboring and non-laboring women, the expression of *CD1D* (Cluster of differentiation 1 D) encoding for the non-classical major histocompatibility complex (MHC) CD1d implicated in invariant NK T (iNKT) cell activation, was upregulated in preterm laboring women, implying different iNKT activation status in preterm labor (221, 225). Understandably, high inter-patient variability presents challenges in characterizing decidual immune cell populations in term versus preterm labor, as seen by various conflicting results (221).

##### 3.1.3.3 Decidual hemorrhage

Decidual hemorrhage, either as vaginal bleeding or retroplacental hematoma formation, is also implicated in sPTB. Abnormal bleeding from placental abruption, poorly transformed spiral artery, and/or defective decidual hemostasis allows decidual tissue factors to initiate thrombin production (226). Thrombin then binds to protease-activated receptors PAR1 and PAR3 to stimulate myometrial contractility (227), upregulate MMP expression (228), inhibit decidual nuclear progesterone receptor expression (226), and induce decidual IL-8 production leading to sterile neutrophil infiltration in the decidua (229).

#### 3.1.4 Summary

**Figure 3:**
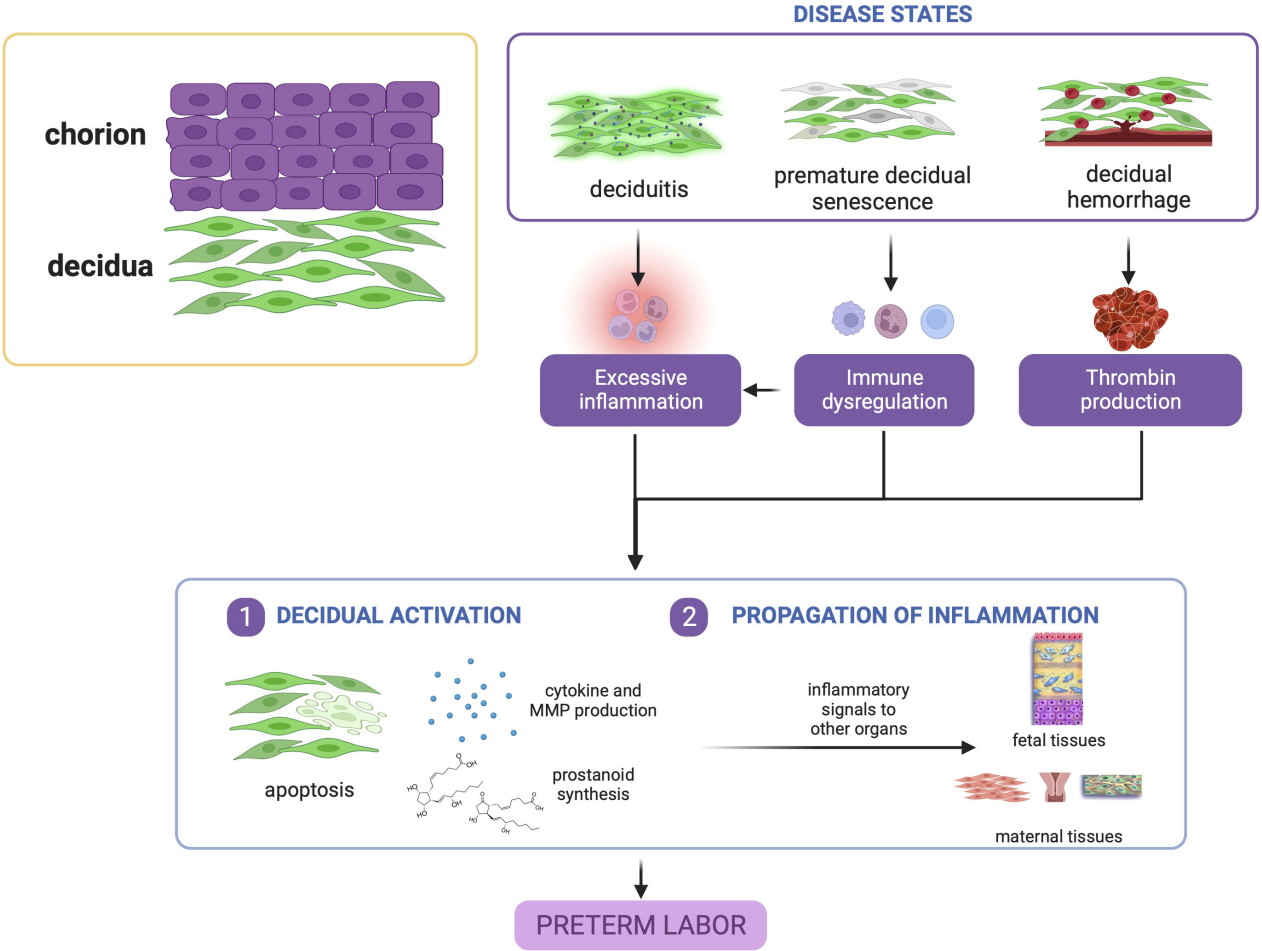
Disease states in the decidua contribute to preterm birth. Deciduitis, decidual hemorrhage, and premature decidual senescence all eventually converge towards premature decidual activation characterized by leukocyte activation, inflammatory mediator production, and decidual apoptosis. This figure was created with BioRender.com.

In summary, evidence points to the decidua as a vital tissue mediating the onset of labor *via* decidual activation. Decidual activation is primarily enhancement of inflammation, where resident immune cells and immune cell-like decidual cells contribute to a dysregulation of decidual inflammatory homeostasis before term leading to PTB, as shown in **Figure 3**.

### 3.2 Preterm birth: A disease of the myometrium

#### 3.2.1 Structure and function of the myometrium during pregnancy

The uterus undergoes substantial remodeling and adaptation throughout the course of pregnancy. The myometrium, the middle muscular layer of the uterus, stays quiescent throughout gestation until mechanical and endocrine signals stimulate its coordinated contraction at term culminating in labor and parturition (230, 231). Therefore, it can be imagined that the uterine myometrium serves as another terminal destination for signals from the other feto-maternal compartments that lead to its awakening.

#### 3.2.2 Myometrial contributions in parturition

Functional progesterone withdrawal may allow the myometrium to adopt a contractile phenotype for laboring, either in term or preterm labor (232, 233). Shortly before and during human labor, the PR-A/PR-B mRNA ratio increases, resulting in increased expression of estrogen receptor alpha (ERα) and homeobox A10 (HOXA10) (234). Since progesterone inhibits the expression of ERα and HOXA10, this indicates that functional progesterone withdrawal is linked to increased expression of PR-A relative to PR-B. PR-A expression is promoted by co-factor Krueppel-like factor 9 (KLF-9) and is regulated at the epigenetic and posttranslational levels (235). Jumonji AT-rich interactive domain 1A (JARID1A), a histone H3 lysine 4 (H3K4) demethylase, occupies the promoter of PR-A during gestation, resulting in lower H3K4me3 levels compared to laboring myometrium and transcriptional repression of PR-A (236). Histone deacetylase 1 (HDAC1) was also reported to bind to the PR-A promoter, resulting in decreased PR-A mRNA levels (237). When these epigenetic regulators are downregulated during labor, they increase PR-A levels. Post-translationally, PR-A degradation by proteasomes is decreased in response to IL-1β, thus leading to sustained PR-A levels (43). In addition, there is upregulation of 20α-hydroxysteroid dehydrogenase (20α-HSD) in the placenta of mice and humans which converts progesterone into inactive 20α-dihydroprogesterone (238–240). This causes progesterone to be unbound from the PR-A, resulting in PR-A translocation from the cytoplasm to the nucleus. PR-A then interacts with Fos-related antigen-2 (Fra-2)/Jun heterodimers, another type of activator protein 1 (AP-1) transcription factor, at the promoter region, thus resulting in Cx43 expression (233). These changes have been seen in both term and preterm labor, suggesting that functional progesterone withdrawal may be a joint promoter of labor in preterm and term birth (233).

Contraction that follows progesterone withdrawal supports the classical process ascribed to the myometrium that contributes to labor and parturition. The functional unit of the myometrium, the uterine smooth muscle cell (SMC), orchestrates contraction *via* the expression of CAPs such as oxytocin receptors, connexin 43, and COX-2 (230). The classic hormone implicated in contraction is oxytocin, a nanopeptide produced primarily by the paraventricular nucleus of the anterior pituitary but is also locally produced by the decidua, placenta, and fetal membranes (241). Its cognate receptor, the oxytocin receptor (OXTR), acts *via* phospholipase C (PLC) coupling leading to inositol 1,4,5-triphosphate (IP3) and diacylglycerol (DAG) formation and the canonical calcium deployment from the sarcoplasmic reticulum (241). PR-A stimulation induces the formation of gap junction *via* Cx43 that promotes synchronization of myometrial signals for contraction (242). COX-2 is required for the eventual formation of prostaglandins crucial for a physiological immunological response (243).

Aside from a contractile phenotype, SMCs appear to have other phenotypes as well enriched for neutrophil and IFN-γ response that allows for inflammation sensing within the local environment (244). During early phases of labor, there is no evidence of activation of pro-inflammatory transcription factor NF-κB and no inflammatory cell infiltration of the myometrium, suggesting that myometrial inflammation is a consequence of labor rather than a cause (245). Consequently, during the contractile phase of the myometrial transformation, there is an associated upregulation of pro-inflammatory cytokines (246, 247), cellular adhesion molecules (248, 249), and infiltration of macrophages, leukocytes, and neutrophils (246, 249) leading to activation of NF-κB and downstream signaling, suggesting that uterine inflammation is a physiologic phenomenon in the process of labor (250, 251). Other mediators, such as deglycase DJ-1 encoded by Parkinson protein 7 gene (*PARK7*) (252), nuclear factor erythroid 2–related factor 2 (NRF-2) and heme oxygenase 1 (HO-1) (253), Rho/Rho-associated protein kinase (ROCK) signaling (254), GRK-Interacting Protein 2 (GIT2) (255), dimerization partner, RB-like, E2F and multi-vulval class B (DREAM) complex (256), and neuromedin-U receptor 2 (NmU-R2) (257) have been suggested to play roles as well in propagating inflammation within the myometrium.

In mouse models, it was observed that preterm labor and term labor shared around 60% of similarly regulated genes, with two waves of gene expression changes corresponding to tissue remodeling and contractility activation, respectively (258). Consistent with these findings, myometrial inflammation is relatively rare in preterm labor parturients compared to term parturients (259). Therefore, myometrial inflammation in itself is not a requisite for preterm labor, and so its interplay with contractility as a cyclical feed-forward mechanism is perhaps a crucial process toward sPTB (260, 261).

#### 3.2.3 Disease states in the myometrium contributing to sPTB

##### 3.2.3.1 Progesterone receptor dysregulation

The progesterone and PR signaling is integrated into a regulatory network *via* miRNA targeting of the zinc finger E-box binding homeobox (ZEB) which culminates into the NF-κB pathway. Reduced progesterone signaling leads to a reduction of ZEB1/2, which derepresses the expression of miRNA-200b/429 and results to a feed-forward mechanism that further negatively regulates ZEB1/2 expression (239, 262). This leads to the expression of OXTR and Cx43, which leads to myometrial activation. Other miRNAs have also been explored that may regulate PR, ER, and ZEB expression as well (263, 264). More importantly, epigenetic dysregulations in ZEB1/2 expression as shown *via* a multigenerational stress model suggests that a familial predisposition towards PTB may be a causative mechanism (265). Nonetheless, these miRNAs may serve as local mediators only, as other systemic miRNAs may offer a more predictive function despite the relative paucity of mechanistic evidences for these systemic miRNAs (266, 267).

##### 3.2.3.2 Uterine overdistension

Mechanical forces may also play contributory roles toward a PTB phenotype. Uterine overdistension arising from multifetal gestation, polyhydramnios, large-for-gestational-age pregnancy, and maternal stature disproportionate to uterine load size has also been associated with sPTB and pPROM (11, 268, 269). Contraction-associated proteins such as gap junction proteins Cx43 and Cx26, oxytocin receptors, PGHS-2, and PGE were upregulated in myometria of pregnant rats and primary human uterine myocytes as a result of uterine stretch (270–272). Aside from mechanical signals, the myometrium also takes cues from endocrine signals, given that stretch-induced expression of Cx43 in rat myometria is facilitated by progesterone withdrawal (273). Increased expression of cytokines IL-1β, TNF-α, IL-6, IL-8 and CCL2, and prostaglandins PGE2 and PGF2α was also observed in a non-human primate model of uterine overdistension, which correlated with preterm labor (274). Aside from gap junction proteins and inflammatory molecules, genes involved in tissue remodeling and muscle growth were differentially expressed in both non-human primate models and in pregnant women with either polyhydramnios or twins (274). Specifically, there is increased collagenase activity in the lower uterine segment due to uterine stretch, thus facilitating the ripening of adjacent cervical tissue (193). These findings imply that when there is sufficient uterine stress from overdistension, the myometrium responds with an inflammatory pulse that contributes to early labor initiation (Kumar et al., 2016).

##### 3.2.3.3 Myometrial hemorrhage

Hemorrhages may also trigger myometrial contractions *via* thrombin-mediated signaling (275–277). Although myometrial bleeding is only expected postpartum, this may be important in the setting of other pathologies such as spontaneous bleeding secondary to placenta previa, placenta abruptio, or miscarriages. The coagulation factor thrombin and its associated receptor protease-activated receptor 1 (PAR1) is expressed in myometrial cells circumscribing the hemorrhage, with concomitant upregulation of myosin, COX-2, PGE2, and PGF2α (277). It has been proposed that thrombin-antithrombin III (TAT) complex be utilized as a prognostic marker for preterm delivery. However, this remains to be seen if this can be improved in combination with other biomarkers for increased sensitivity (275).

##### 3.2.3.4 Oxidative stress in the myometrium

Oxidative stress specifically occurring from environmental insults and behavioral risk exposures may lead to PTB *via* induction of inflammation and preterm progesterone withdrawal in the myometrium. Various metal toxicants also increase 8-hydroxyguanosine (8-OHdG), a marker of oxidative stress, and some metals were positively associated with sPTB; however, the exact mechanisms may involve a separate pathway activated by oxidative stress or another factor (278). It is unclear if myometrium is the primary target for toxicants or if the observed changes are secondary to insults from other tissues responding to various exposures. Oxidative stress also induces exosome production in myometrial cells, and these exosomes may be transported to amnion epithelial cells and chorion trophoblast cells resulting in the local production of IL-6 and TNF-α (279). Interestingly, IL-10 was also significantly upregulated in chorion trophoblast cell cultures post-exposure to myometrial exosomes, a counteracting mechanism to provide relief to acute inflammation (171, 279).

#### 3.2.4 Summary

**Figure 4:**
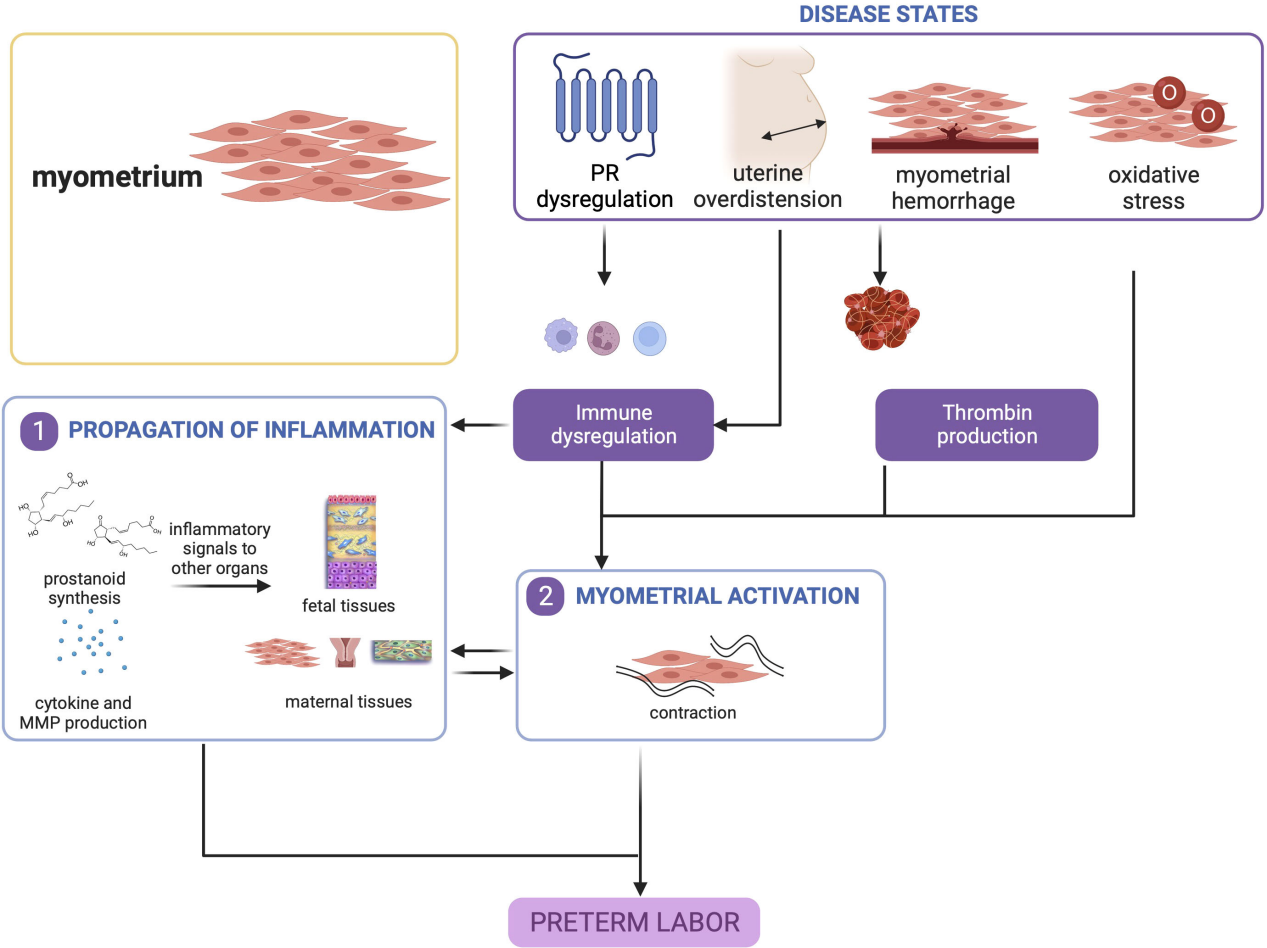
Disease states in the myometrium contribute to preterm birth. Uterine overdistension and myometrial hemorrhage, and to a lesser extent, oxidative stress, may all lead to myometrial activation that produces contractions. This figure was created with BioRender.com.

Overall, myometrial inflammation resulting from uterine overdistension, hemorrhage, and, to a lesser extent oxidative stress, is a key contributor to PTB. It is important to note that myometrial inflammation alone is not a requisite for sPTB, but other concomitant processes in other gestational tissues may tip the balance towards a cascade of signaling that will lead to preterm delivery as shown in **Figure 4**.

### 3.3 Preterm birth: A disease of the cervix

#### 3.3.1 Structure and function of the cervix in pregnancy

Cervix is a firm cylindrical structure located at the lower pole of the uterine corpus (280). It is composed of the cervical epithelial layer (ectocervix, transformation zone, and endocervix), which protects the underlying cervical stromal layer formed of mesenchymal cells, smooth muscle cells, immune cells, and ECM proteins such as collagen and elastin (280–283). ECM proteins in the stromal layer and the cervical smooth muscle cells provide robust rigidity (284–286). The cervical epithelial layer contains junction proteins that seal intercellular space between adjacent cells and maintain apical and basolateral polarity (287, 288). The endocervical epithelial cells also produce a mucus plug that further seals the cervical canal (289, 290). As such, the cervix serves as a barrier protecting the developing fetus in the uterine cavity from potential pathogens present in the vaginal canal (289, 291, 292). Additionally, antimicrobial peptides such as cathelicidin, elafin, human β-defensin-3, and human neutrophil elastase are also expressed in the cervical mucus (293–295). The cervix can also mount an immune and inflammatory response to prevent ascending infection, *via* upregulation of IL-1β, IL-6, and IL-8 in the cervical mucus (295–298). Counter-regulatory measures to prevent massive inflammation also exists *via* the expression of IL-4, IL-10, and IL-13 (296, 299, 300).

#### 3.3.2 Cervical processes in parturition

As the pregnancy progresses to labor, the cervix undergoes gradual remodeling and softening, and during parturition, it ripens and dilates to allow safe passage of the fetus in combination with uterine contractions (301, 302). Several underlying mechanisms are associated with the cervical remodeling process. Some of the known mechanism involves an influx of immune cells such as neutrophils and macrophages (247, 303), an increase in inflammatory mediators including cytokines (296, 301, 303–305), activation of NF-κB, platelet-activating factor, and prostaglandin synthases COX-1 and COX-2 (305, 306). Other mechanisms such as changes in steroid hormone receptors (71, 307–309), increase in oxidative stress and derangements in antioxidant enzymes (310–313), apoptosis and necrosis (309, 313–315), and epithelial-to-mesenchymal transition in resident cervical cell types (315–319) have also been reported. The mechanical stretch from the developing fetus also increases pressure, reduces blood supply, and promotes hypoxia in the cervix. Elevated levels of hypoxia-inducible factor-1α (HIF-1α) in the cervix promote cervical ripening and dilation *via* inflammation in a feed-forward mechanism (281, 282, 320).

Dynamic changes in ECM proteins, mainly collagen, decorin, elastin, glycosaminoglycans, and hyaluronic acid, occur in cervical remodeling in labor (302, 321–324). Proteomic studies have shown that highly cross-linked collagens were replaced by newer, less cross-linked collagen during parturition (324), resulting in increased tissue compliance in cervical ripening. Aside from collagen, hyaluronic acid is essential due to its role as a component of the ECM. Loss of hyaluronan synthesis may also alter cell transitions among cervical epithelial cells (325). As demonstrated in a mouse model, knockout of hyaluronan synthase leads to the cycling of basal goblet cells with concomitant intracellular sequestering of Olfm4 and a striking loss of Serpina1e proteins, which are proteins that limit inflammatory processes in target tissues (326, 327). Overall, hyaluronan loss decreases the immunoprotective barrier function provided by the cervix, increasing vulnerability to ascending infection and, consequently, preterm birth (325, 328). However, these mechanisms need to be further studied in humans.

Steroid hormones, such as estrogen and progesterone, play a key role in regulating the timing of cervical remodeling (307–309, 316, 322, 329). The local levels of these hormones influence cervical collagen and elastic fiber synthesis, assembly, and turnover, influencing ripening (322). During pregnancy, 17β-hydroxysteroid dehydrogenase (17β-HSD) production by glandular epithelial cells increases and catalyzes the conversion of estradiol to estrone and cervical stroma-derived 20α-hydroxyprogesterone to progesterone (330). Progesterone upregulates nitric oxide production in the uterus, promoting uterine quiescence, and downregulates nitric oxide production in the cervix, maintaining suitable cervical rigidity to maintain pregnancy (305, 331–333). During parturition, 17β-HSD2 is downregulated in endocervical cells, increasing estrogen levels and creating a favorable microenvironment for cervical ripening. In addition, functional progesterone withdrawal *via* PR-A preferential expression may also contribute to cervical ripening, since it was demonstrated that an increase in PR-A isoform in the cervical stromal cells promotes a local withdrawal of progesterone effects (307, 308). Any aberration in this physiological process may lead to adverse birth outcomes such as miscarriage or sPTB (283, 334, 335).

#### 3.3.3 Disease states of the cervix contributing to sPTB

##### 3.3.3.1 Cervical insufficiency and short cervix

Cervical insufficiency resulting in a short cervix is a leading cervical cause of preterm birth. This pathology can be either congenital, as a consequence of Ehlers-Danlos syndrome, or Marfan syndrome, but may also be secondary to non-syndromic forms of mild collagenopathies (336). This may also be an acquired condition, as those occurring from cervical trauma such as cervical lacerations from childbirth (337, 338) and previous cervical surgery such as conization and loop electrosurgical excision procedure (291, 339, 340). In the absence of a bacterial etiology, cervical insufficiency leading to a short cervix can be thought of as a consequence of premature cervical ripening, which occurs *via* upregulation of inflammation in the preterm period (341). Pathological states that lead to abrupt progesterone withdrawal have been proposed as a mechanism for this phenomenon leading to a decrease in collagen stability. Other non-leukocyte-dependent mechanisms such as complement activation, macrophage-mediated MMP release, and 15-prostaglandin dehydrogenase inhibition may also contribute to its pathophysiology (88, 341, 342). Nevertheless, more studies are needed in order to establish a non-infectious etiology for cervical insufficiency.

Another potential cause of cervical insufficiency is the presence of a dysregulated cervicovaginal microbiota. Multiple studies have demonstrated that an abnormal microbiota is positively associated with a shortened cervix and PTB, especially in situations wherein *Lactobacillus* dominance is disrupted and is predominated by both aerobic and anaerobic bacteria (343–345). Aerobic vaginitis and *Mycoplasma hominis* were also associated with cervical shortening, although their presence is not requisite in bringing about PTB (343). An abnormal microbiota may have implications for cervical remodeling leading to a short cervix, but studies need to be carried out to provide definitive evidence for this.

Regardless of etiology, cervical insufficiency may potentially result to (1) a weakened epithelium that favorably allows cervicovaginal microbes to break through the cervical barrier and promote ascending infection, and/or (2) compromised cervical support that increases the pressure on the fetal membranes and potentially exposes them to the external environment *via* an open internal os (334, 346–349). Both processes can lead to downstream inflammatory responses that trigger sPTB.

##### 3.3.3.2 Cervicitis

Cervical infections have been shown as risk factors for prematurity and is an independent risk factor for PTB for some infectious agents, irrespective of treatment status (291). Inflammation secondary to infection may alter the cervical epithelial barrier *via* actions of pro-inflammatory cytokines (315, 350, 351).

Since the cervix does not operate in silo, propagation of localized inflammation in the cervix may impact the upper gestational tissues as well and result to activation of inflammatory cascades. These inflammatory signatures have been recently shown to be carried out *via* paracrine signaling *via* extracellular vesicles (EV), such as exosomes and microvesicles. EVs also serve as a mechanism for intercellular interaction of the cervix with other feto-maternal cells. Cervical cells can also produce exosomes that elicit an inflammatory response in feto-maternal interface tissues that promote parturition and vice-versa (140, 279, 352, 353).

##### 3.3.3.3 Conditions leading to a weakened cervical epithelium

Despite the presence of multiple defense mechanisms, commensal and pathogenic bacteria and viruses can still compromise the cervix, ascend into the amniotic cavity, infect the fetus, and promote fetal-maternal inflammation and sPTB (291, 354–357). Any condition that can compromise the epithelial barrier of the cervix can allow for potential invasion from these microbes. Damage to the cervical mucosa secondary to chemical (e.g., irritants) or mechanical (e.g., cervical trauma from lacerations, abrasions, or previous surgeries such as conization and excision procedures) predisposes the cervix to infection and increases PTB risk (292, 337, 338, 358–360). Epigenetic changes induced by microRNAs may also contribute to a decrease in the tightness of the epithelial barrier (361).

Ascending infection may be established *via* direct seeding; however, recent studies have shown that cervical exosomes could transport intracellular bacteria (e.g., *Chlamydia trachomatis* and *Ureaplasma parvum*) and bacterial proteins from infected cells to uninfected cells. This can be a possible mechanism for breaching the cervical barrier and establishing ascending infection to the amniotic cavity (362, 363). Supracervical infections in the decidua, fetal membranes, and less commonly in the placenta, can lead to localized inflammation depending on the location, and lead to inflammatory processes as aforementioned.

#### 3.3.4 Summary

**Figure 5:**
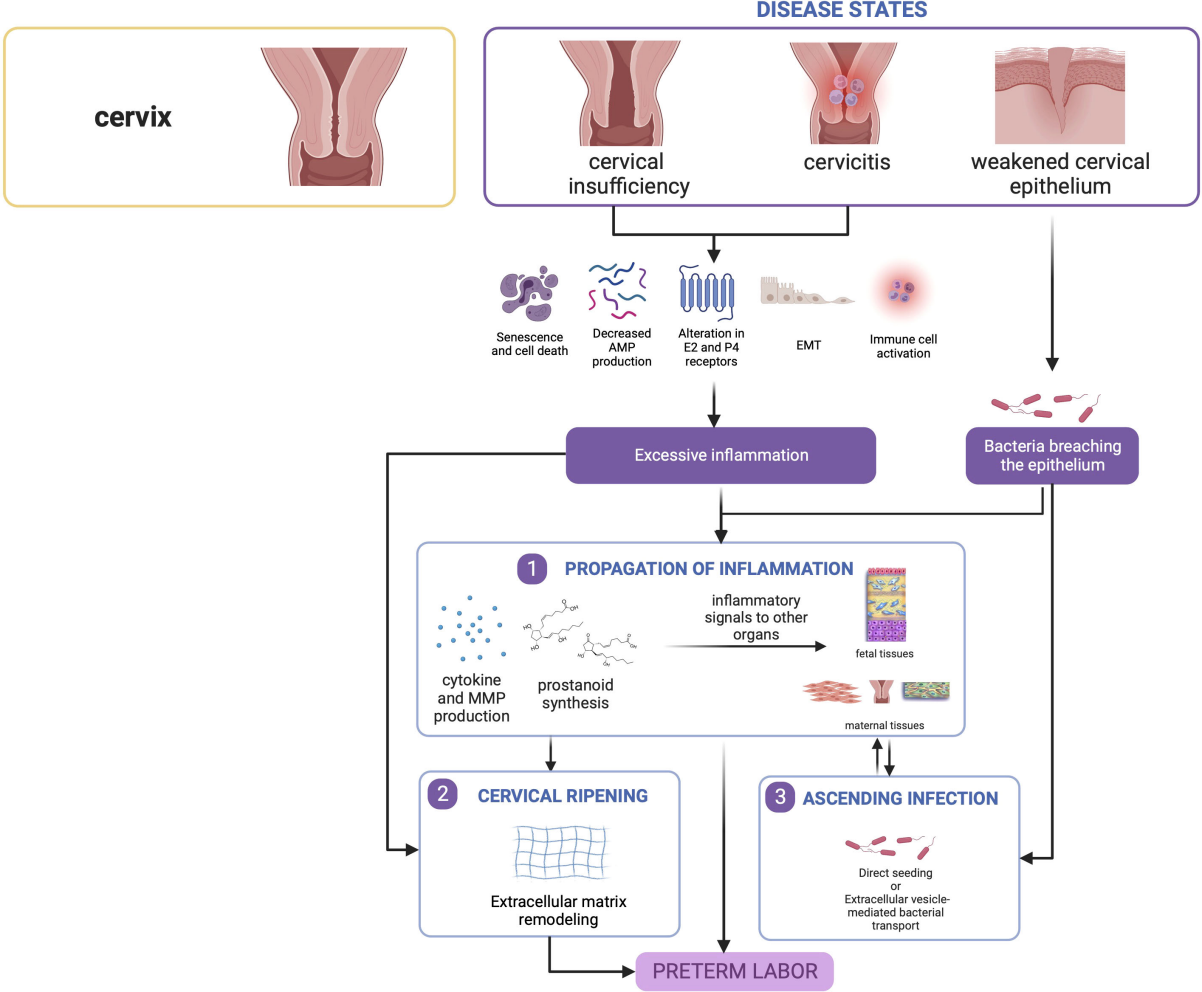
Disease states in the cervix contribute to preterm birth. Cervical insufficiency and cervicitis may lead to localized inflammatory responses that can propagate towards other tissues. Similarly, a weakened epithelium may allow passage of bacteria leading to ascending infection, which also causes inflammation in seeded gestational tissues. This figure was created with BioRender.com.

Overall, inflammatory processes govern cervical contributions toward PTB. The cascade of inflammation may either result from infection secondary to a compromised cervical barrier, or potentially from sterile premature cervical ripening secondary to mechanisms yet to be elucidated as shown in **Figure 5**.

## 4 Discussion and conclusions

Studying each tissue and organ system has educated us on their disease states and respective contributions to generating pathways leading to PTB. Current preventive interventions that are partly effective in a small subset of high-risk populations include progesterone (possibly targeting PR dysfunction and inflammation in the myometrium, placenta, and cervix), and cerclage and pessary application (targeting cervical insufficiency) (364, 365); however, these approaches cannot be simply administered to the general population and are not universal intervention strategies. In the context of active uterine contractions, various pharmacologic agents have been explored as therapeutics, including beta-adrenergic receptor agonists, calcium channel blockers, magnesium sulfate, COX inhibitors, nitric oxide donors, and oxytocin receptor antagonists (366). However, terminating uterine contractions does not equate to resolving preterm delivery, and patients may still deliver prematurely even with tocolytic agents (366). In summary, extensive knowledge on various organs have led us to develop each organ system based intervention strategies to reduce the risk of sPTB; however, this approach alone has not reduced sPTB rates because each organ system generate their own unique mechanistic pathways in response to various exposures but they also impact other tissues to react and respond. So, a systemwide knowledge is required to effectively mitigate the risk of sPTB.

**Figure 6:**
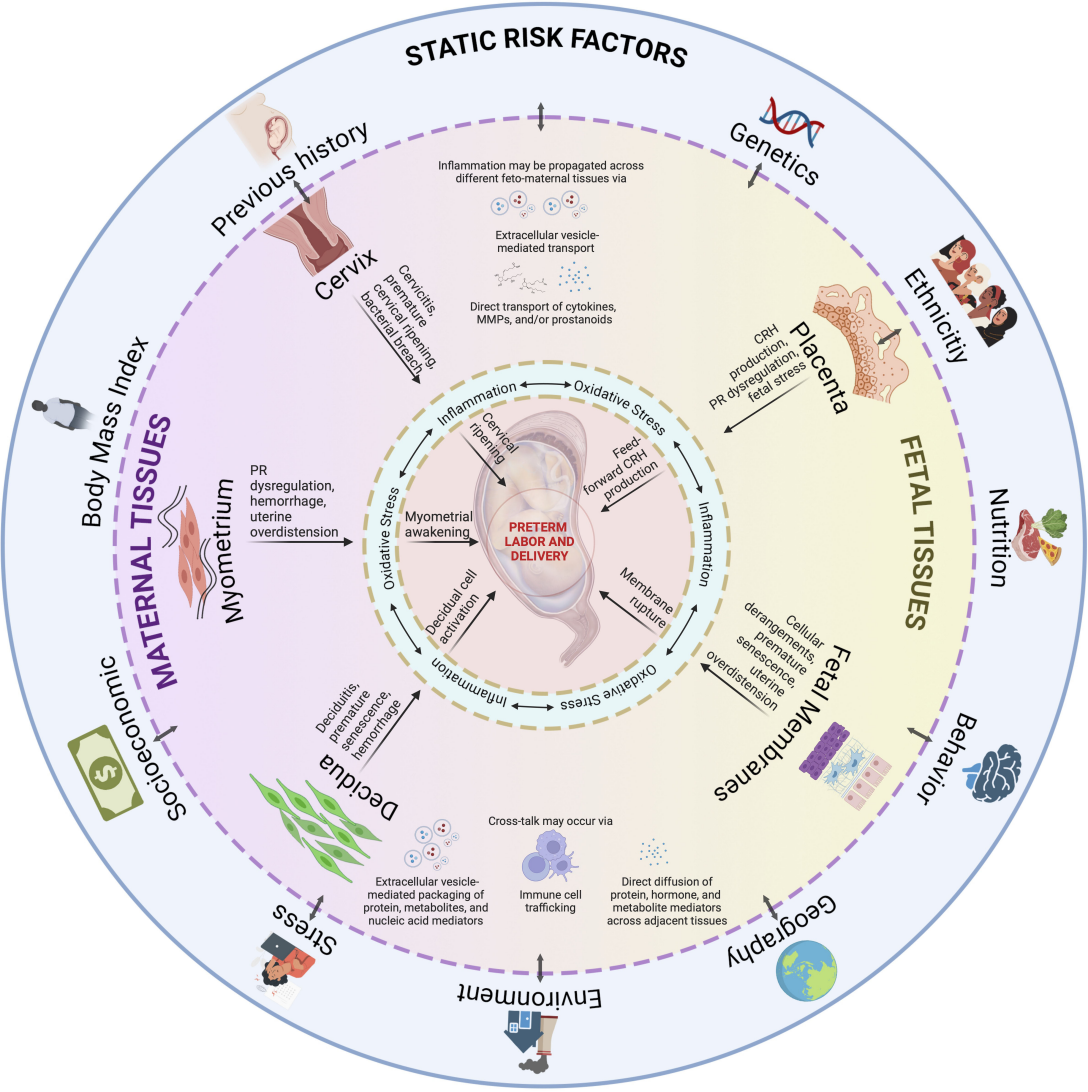
An integrated model for PTB. Static risk factors may provide susceptibility to inherited dysregulations in the fetal and maternal tissues. An interaction with ‘pregnancy environment’ can contribute to dynamic risk factors, such as those from acquired disease states as discussed per tissue subsection, may occur throughout the pregnancy. Inflammation, and oxidative stress, provides the central tenet for PTB as they are inseparable in this process. As inflammation may propagate across different feto-maternal tissues, and tissue cross-talk may occur *via* various mechanisms, multiple contributions from affected tissues will eventually culminate to PTB. PTB is not a disease of a single system acting in silo but contributed by systems working together to produce an outcome. This figure was created with BioRender.com.

However, it is also obvious that tissue and organ-specific knowledge alone is insufficient to fully understand sPTB, or even develop biomarkers to predict sPTB at an early stage. Although we have tissue- and organ-specific interventions, these are relegated to specific indications only; thus, there is still a need to develop management strategies to definitively reduce risk and, potentially, provide a cure for sPTB. Investigators have associated *in vitro* model findings (2D cell culture, trans well multi-cell systems, and explant culture systems) and developed multisystem prediction models over the years, primarily explaining how one mechanistic event in a cell or tissue may lead to a sPTB pathway in another tissue as we described above. Many of the mechanistic events in each system have been validated using animal models confirming data related to a specific tissue/organ system and highlighted limitations (367–369). The sustained rate of sPTB suggests that current studies examining a single system (maternal or fetal) or translating cell culture data into animals do not always reflect sPTB pathophysiology. An integrated model that can incorporate all the interactions between various systems as seen *in utero *(**Figure 6**) is difficult to replicate in real-time using current approaches.

Fortunately, recent advances have been developed that allow for the incorporation of multiple cell lines from various systems to simulate a live organ using microphysiological systems. Using cells, matrix, immune system, and vasculature from a source tissue or organ, and placing these on an organ-on-a-chip (OOC), a biomimetic microfluidic system, researchers are recreating an organ’s architecture *in vitro* (370). These integrated OOC models allow simultaneously testing the responses and interactions between multiple systems. Various OOC models have already been developed, including a human-on-a-chip that has incorporated various organ systems including the heart, liver, lungs, gut, and kidney (371). Understanding PTB, developing biomarkers, and testing new drugs require an integrated model system where feto-maternal units can communicate and generate coordinated responses as expected *in utero* (370, 372). Several OOC models are being developed and are currently being tested in the reproductive biology field, and we concur that the next decade likely will shift from using traditional model systems to integrated chip models utilizing human tissues and cells (373). OOC models developed are now being used for preclinical testing and their utility has been recognized by various regulatory agencies that are demanding the reduction of unnecessary use of animal models in such studies (370). Hopefully, in the nearest future, these advances can address the lack of reliable and cost-effective animal models to evaluate potential pharmacologic interventions for preterm birth and elucidate molecular mechanisms closer to the human pathophysiology. This will reduce the use of animal models, improve our knowledge of pregnancy and parturition, and reduce the incidences of preterm parturition.

## Author contributions

RM – conceptualization and supervision. RM, MV, and MS – visualization. MV, RL, MS, and OT – writing the original draft. MV, RL, and RM – review and editing of the subsequent drafts. All authors contributed to the article and approved the submitted version.

## Funding

This study is funded *via* the NIH/NICHD Grant 5R01HD100729-03 to RM.

## Acknowledgments

MV, RL, MS and OT are current and past MD-PhD in Molecular Medicine Program trainees supported by the Department of Science and Technology - Philippine Council for Health Research and Development (DOST-PCHRD). We would like to thank Nina Truong of UTMB at Galveston for lending her talent in illustrating the center piece of the summary figure for this article.

## Conflict of interest

The authors declare that the research was conducted in the absence of any commercial or financial relationships that could be construed as a potential conflict of interest.

## Publisher’s note

All claims expressed in this article are solely those of the authors and do not necessarily represent those of their affiliated organizations, or those of the publisher, the editors and the reviewers. Any product that may be evaluated in this article, or claim that may be made by its manufacturer, is not guaranteed or endorsed by the publisher.

## Abbreviations

11β-HSD1 11-beta hydroxysteroid dehydrogenase 1; 15-HPGD 15-hydroxyprostaglandin dehydrogenase; 17β-HSD 17β-hydroxysteroid dehydrogenase; 20α-HSD 20α-hydroxysteroid dehydrogenase; 8-OHdG 8-hydroxyguanosine; AEC Amnion epithelial cells; AFTR2 Alternaria alternata AF-toxin gebe cluster; AMP Antimicrobial peptide; AP-1 Activator protein-1 CAP Contraction-associated protein; CCL4 Chemokine (C-C ; CD1D motif) ligand 4 Cluster of differentiation 1 D; cffTF Cell-free fetal telomere fragments; COX-2 Cyclooxygenase -2; CRH Corticotropin-releasing hormone; CT Cytotrophoblast; Cx43 Connexin 43; DAG Diacylglycerol; DAMP Damage-associated molecular patterns; DREAM Dimerization partner, RB-like, E2F and multi-vulval class B; EBF1 EBF transcription factor 1; ECM Extracellular matrix; EEFSEC Eukaryotic Elongation Factor, Selenocysteine-TRNA Specific; EMT Epithelial-to-mesenchymal transition; ER Estrogen receptor; ESC Endometrial stromal cell; EV Extracellular vesicles; EVT Extravillous trophoblast; GIT2 GRK-Interacting Protein 2; GWAS Genome-wide association studies; H3K4 Histone H3 lysine 4; HAND2 Heart And Neural Crest Derivatives Expressed 2; Hcg Human chorionic gonadotropin; HDAC1 Histone deacetylase 1; HIF-1α Hypoxia-inducible factor-1α; HMGB1 High mobility group box 1; HO-1 Heme oxygenase 1; HOXA10 Homeobox A10; hPL Human placental lactogen; HSP-70 Heat schock protein-70; IGFBP1 Insulin-like growth factor-binding protein 1; IL Interleukin; iNKT Invariant natural killer T; IP3 Inositol 1,4,5-triphosphate; JARID1A Jumonji AT-rich interactive domain 1A; KLF-9 Krueppel-like factor 9; LPS Lipopolysaccharide; MAPK Mitogen-activated protein kinase; MET Mesenchymal-to-epithelial transition; MHC Major histocompability complex; MIP-1β Macrophage inflammatory protein (MIP)-1β; MMP Matrix metalloproteinase; mTORC1 Mammalian target of rapamycin complex 1-; NF-kB Nuclear factor kappa B; NIK NF-kB inducing kinase; NmU-R2 Neuromedin-U receptor 2; NRF-2 Nuclear factor erythroid 2–related factor 2; OOC Organ-on-a-chip; OXTR Oxytocin receptor; PAF Platelet-activating factor; PAR Protease-activated receptor; PARK7 Parkinson protein 7; PGDH Prostaglandin dehydrogenase; PGE2 Prostaglandin E2; PGF2α Prostaglandin F2 alpha; PGHS2 Prostaglandin H synthase 2; PGRMC Progesterone receptor membrane component; PI3K Phosphatidylinositol-3 kinase; PLC Phospholipase C; PR Progesterone receptor; PTB Preterm birth; PTEN Phosphatase and tensin homolog; ROCK Rho/Rho-associated protein kinase; SASP Senescence-associated secretory phenotypes; SCT Syncytiotrophoblast; SMC Smooth muscle cell; sPTB Spontaneous preterm birth; TAT Thrombin-antithrombin III; TLR4 Toll-like receptor 4; TNF-α Tumor necrosis factor alpha; uNK Uterine natural killer; VEGF Vascular endothelial growth factor; ZEB Zinc finger E-box binding homeobox

## References

[1] Mor G Aldo P Alvero AB. The unique immunological and microbial aspects of pregnancy. Nat Rev Immunol (2017) 17:469–82. doi: 10.1038/nri.2017.64 28627518

[2] Romero R Dey SK Fisher SJ. Preterm labor: One syndrome, many causes. Science (2014) 345:760–5. doi: 10.1126/science.1251816 PMC4191866 25124429

[3] Spencer TE Bazer FW. Conceptus signals for establishment and maintenance of pregnancy. Reprod Biol Endocrin (2004) 2:49. doi: 10.1186/1477-7827-2-49 PMC471568 15236653

[4] Abu-Raya B Michalski C Sadarangani M Lavoie PM. Maternal immunological adaptation during normal pregnancy. Front Immunol (2020) 11:575197. doi: 10.3389/fimmu.2020.575197 33133091 PMC7579415

[5] Green ES Arck PC. Pathogenesis of preterm birth: bidirectional inflammation in mother and fetus. Semin Immunopathol (2020) 42:413–29. doi: 10.1007/s00281-020-00807-y PMC7508962 32894326

[6] Makino S Zaragoza DB Mitchell BF Yonemoto H Olson DM. Decidual activation: Abundance and localization of prostaglandin F2α receptor (FP) mRNA and protein and uterine activation proteins in human decidua at preterm birth and term birth. Placenta (2007) 28:557–65. doi: 10.1016/j.placenta.2006.06.010 16911823

[7] Leimert KB Xu W Princ MM Chemtob S Olson DM. Inflammatory amplification: A central tenet of uterine transition for labor. Front Cell Infect Mi (2021) 11:660983. doi: 10.3389/fcimb.2021.660983 PMC8417473 34490133

[8] Challis JRG. CRH, a placental clock and preterm labour. Nat Med (1995) 1:416–6. doi: 10.1038/nm0595-416 7585086

[9] Menon R. Preterm birth: a global burden on maternal and child health. Pathog Glob Health (2013) 106:139–40. doi: 10.1179/204777312x13462106637729 PMC4001570 23265368

[10] Menon R. Spontaneous preterm birth, a clinical dilemma: Etiologic, pathophysiologic and genetic heterogeneities and racial disparity. Acta Obstet Gyn Scan (2008) 87:590–600. doi: 10.1080/00016340802005126 18568457

[11] Behrman RE Butler AS eds. Outcomes I of m (US) c on UPB and AH. In: Preterm birth. Washington (DC: National Academies Press (US. doi: 10.17226/11622 20669423

[12] Jaremek A Jeyarajah MJ Bhattad GJ Renaud SJ. Omics approaches to study formation and function of human placental syncytiotrophoblast. Front Cell Dev Biol (2021) 9:674162. doi: 10.3389/fcell.2021.674162 34211975 PMC8240757

[13] Pollheimer J Vondra S Baltayeva J Beristain AG Knöfler M. Regulation of placental extravillous trophoblasts by the maternal uterine environment. Front Immunol (2018) 9:2597. doi: 10.3389/fimmu.2018.02597 30483261 PMC6243063

[14] Knöfler M Haider S Saleh L Pollheimer J Gamage TKJB James J. Human placenta and trophoblast development: key molecular mechanisms and model systems. Cell Mol Life Sci (2019) 76:3479–96. doi: 10.1007/s00018-019-03104-6 PMC6697717 31049600

[15] Burton GJ Jauniaux E. Development of the human placenta and fetal heart: Synergic or independent? Front Physiol (2018) 9:373. doi: 10.3389/fphys.2018.00373 29706899 PMC5906582

[16] Costa MA. The endocrine function of human placenta: an overview. Reprod BioMed Online (2016) 32:14–43. doi: 10.1016/j.rbmo.2015.10.005 26615903

[17] Vacher C-M Lacaille H O’Reilly JJ Salzbank J Bakalar D Sebaoui S. Placental endocrine function shapes cerebellar development and social behavior. Nat Neurosci (2021) 24:1392–401. doi: 10.1038/s41593-021-00896-4 PMC8481124 34400844

[18] Robbins JR Skrzypczynska KM Zeldovich VB Kapidzic M Bakardjiev AI. Placental syncytiotrophoblast constitutes a major barrier to vertical transmission of listeria monocytogenes. PLoS Pathog (2010) 6:e1000732. doi: 10.1371/journal.ppat.1000732 20107601 PMC2809766

[19] Dudley DJ. Immunoendocrinology of preterm labor: The link between corticotropin-releasing hormone and inflammation. Am J Obstet Gynecol (1999) 180:S251–6. doi: 10.1016/s0002-9378(99)70711-8 9914628

[20] Mancuso RA Schetter CD Rini CM Roesch SC Hobel CJ. Maternal prenatal anxiety and corticotropin-releasing hormone associated with timing of delivery. Psychosom Med (2004) 66:762–9. doi: 10.1097/01.psy.0000138284.70670.d5 15385704

[21] Petraglia F Imperatore A Challis JRG. Neuroendocrine mechanisms in pregnancy and parturition. Endocr Rev (2010) 31:783–816. doi: 10.1210/er.2009-0019 20631004

[22] Warren WB Silverman AJ. Cellular localization of corticotrophin releasing hormone in the human placenta, fetal membranes and decidua. Placenta (1995) 16:147–56. doi: 10.1016/0143-4004(95)90003-9 7792278

[23] Gravanis A. Corticotropin releasing hormone CRH in normal and pregnant uterus physiological implications. Front Biosci (1996) 1:e1–8. doi: 10.2741/a137 9159239

[24] Wetzka B Sehringer B Schäfer W Biller S Hör C Benedek E. Expression patterns of CRH And CRH binding protein in human gestational tissue at term. Exp Clin Endocr Diabetes (2003) 111:154–61. doi: 10.1055/s-2003-39778 12784189

[25] Wu X Shen H Yu L Peng M Lai W-S Ding Y-L. Corticotropin-releasing hormone activates connexin 43 *via* activator protein-1 transcription factor in human myometrial smooth muscle cells. Am J Physiol-endoc M (2007) 293:E1789–94. doi: 10.1152/ajpendo.00249.2007 17895291

[26] Tyson EK Smith R Read M. Evidence that corticotropin-releasing hormone modulates myometrial contractility during human pregnancy. Endocrinology (2009) 150:5617–25. doi: 10.1210/en.2009-0348 19846610

[27] You X Gao L Liu J Xu C Liu C Li Y. CRH activation of different signaling pathways results in differential calcium signaling in human pregnant myometrium before and during labor. J Clin Endocrinol Metab (2012) 97:E1851–61. doi: 10.1210/jc.2011-3383 22869609

[28] You X Liu J Xu C Liu W Zhu X Li Y. Corticotropin-releasing hormone (CRH) promotes inflammation in human pregnant myometrium: The evidence of CRH initiating parturition? J Clin Endocrinol Metab (2014) 99:E199–208. doi: 10.1210/jc.2013-3366 24248185

[29] Jones SA Challis JRG. Effects of corticotropin-releasing hormone and adrenocorticotropin on prostaglandin output by human placenta and fetal membranes. Gynecol Obstet Inves (1990) 29:165–8. doi: 10.1159/000293368 2162798

[30] Alvi SA Brown NL Bennett PR Elder MG Sullivan MHF. Corticotrophin-releasing hormone and platelet-activating factor induce transcription of the type-2 cyclo-oxygenase gene in human fetal membranes. Mhr Basic Sci Reprod Med (1999) 5:476–80. doi: 10.1093/molehr/5.5.476 10338371

[31] Zoumakis E Margioris AN Stournaras C Dermitzaki E Angelakis E Makrigiannakis A. Corticotrophin-releasing hormone (CRH) interacts with inflammatory prostaglandins and interleukins and affects the decidualization of human endometrial stroma. Mhr Basic Sci Reprod Med (2000) 6:344–51. doi: 10.1093/molehr/6.4.344 10729317

[32] Karteris E Grammatopoulos D Dai Y Olah KB Ghobara TB Easton A. The human placenta and fetal membranes express the corticotropin-releasing hormone receptor 1α (CRH-1α) and the CRH-c variant receptor. J Clin Endocrinol Metab (1998) 83:1376–9. doi: 10.1210/jcem.83.4.4705 9543169

[33] Jones SA Challis JRG. Local stimulation of prostagiandin production by corttcotropin-releasing hormone in human fetal membranes and placenta. Biochem Bioph Res Co (1989) 159:192–9. doi: 10.1016/0006-291x(89)92422-4 2784314

[34] Karalis K Goodwin G Majzoub JA. Cortisol blockade of progesterone: A possible molecular mechanism involved in the initiation of human labor. Nat Med (1996) 2:556–60. doi: 10.1038/nm0596-556 8616715

[35] Patel FA Funder JW Challis JRG. Mechanism of Cortisol/Progesterone antagonism in the regulation of 15-hydroxyprostaglandin dehydrogenase activity and messenger ribonucleic acid levels in human chorion and placental trophoblast cells at term. J Clin Endocrinol Metab (2003) 88:2922–33. doi: 10.1210/jc.2002-021710 12788907

[36] Taylor AH McParland PC Taylor DJ Bell SC. The cytoplasmic 60 kDa progesterone receptor isoform predominates in the human amniochorion and placenta at term. Reprod Biol Endocrinol Rb E (2009) 7:22–2. doi: 10.1186/1477-7827-7-22 PMC2669089 19284643

[37] Ziyan J Huaibin R Xiaotian M Guangtong S Xiaoqing C Zijiang D. Regulation of progesterone receptor a and b expression in human preterm, term, and postterm placental villi. Acta Obstet Gyn Scan (2010) 89:705–11. doi: 10.3109/00016341003623761 20423281

[38] Zachariades E Foster H Goumenou A Thomas P Rand-Weaver M Karteris E. Expression of membrane and nuclear progesterone receptors in two human placental choriocarcinoma cell lines (JEG-3 and BeWo): Effects of syncytialization. Int J Mol Med (2011) 27:767–74. doi: 10.3892/ijmm.2011.657 21455559

[39] Wang B Parobchak N Rosen T. RelB/NF-κB2 regulates corticotropin-releasing hormone in the human placenta. Mol Endocrinol (2012) 26:1356–69. doi: 10.1210/me.2012-1035 PMC5416986 22734038

[40] Wang B Palomares K Parobchak N Cece J Rosen M Nguyen A. Glucocorticoid receptor signaling contributes to constitutive activation of the noncanonical NF-κB pathway in term human placenta. Mol Endocrinol (2013) 27:203–11. doi: 10.1210/me.2012-1309 PMC5417329 23239753

[41] Wang B Parobchak N Rosen M Roche N Rosen T. Negative effects of progesterone receptor isoform-a on human placental activity of the noncanonical NF-κB signaling. J Clin Endocrinol Metab (2014) 99:E320–8. doi: 10.1210/jc.2013-2721 24276461

[42] Palomares K Wang B Parobchak N Rosen T. 77: Epigenetic regulation of progesterone receptor isoform-a (PR-a) in the human placenta. Am J Obstet Gynecol (2016) 214:S56. doi: 10.1016/j.ajog.2015.10.096

[43] Peters GA Yi L Skomorovska-Prokvolit Y Patel B Amini P Tan H. Inflammatory stimuli increase progesterone receptor-a stability and transrepressive activity in myometrial cells. Endocrinology (2016), 158(1): en.2016–1537. doi: 10.1210/en.2016-1537 PMC5412979 27886516

[44] Tan H Yi L Rote NS Hurd WW Mesiano S. Progesterone receptor-a and -b have opposite effects on proinflammatory gene expression in human myometrial cells: implications for progesterone actions in human pregnancy and parturition. J Clin Endocrinol Metab (2012) 97:E719–30. doi: 10.1210/jc.2011-3251 PMC3339884 22419721

[45] Zeldovich VB Bakardjiev AI. Host defense and tolerance: Unique challenges in the placenta. PLoS Pathog (2012) 8:e1002804. doi: 10.1371/journal.ppat.1002804 22912572 PMC3415450

[46] Cao B Macones C Mysorekar IU. ATG16L1 governs placental infection risk and preterm birth in mice and women. JCI Insight (2016) 1:e86654. doi: 10.1172/jci.insight.86654 28018968 PMC5161251

[47] Lamont RF Sobel J Mazaki-Tovi S Kusanovic JP Vaisbuch E Kim SK. Listeriosis in human pregnancy: a systematic review. J Perinat Med (2011) 39:227–36. doi: 10.1515/jpm.2011.035 PMC3593057 21517700

[48] Nielsen SY Mølbak K Henriksen TB Krogfelt KA Larsen CS Villumsen S. Adverse pregnancy outcomes and coxiella burnetii antibodies in pregnant women, Denmark - volume 20, number 6–June 2014 - emerging infectious diseases journal - CDC. Emerg Infect Dis (2014) 20:925–31. doi: 10.3201/eid2006.130584 PMC4036770 24856281

[49] Alsaif M Dabelah K Featherstone R Robinson JL. Consequences of brucellosis infection during pregnancy: A systematic review of the literature. Int J Infect Dis (2018) 73:18–26. doi: 10.1016/j.ijid.2018.05.023 29885371

[50] Arora N Sadovsky Y Dermody TS Coyne CB. Microbial vertical transmission during human pregnancy. Cell Host Microbe (2017) 21:561–7. doi: 10.1016/j.chom.2017.04.007 PMC6148370 28494237

[51] Steinborn A Niederhut A Solbach C Hildenbrand R Sohn C Kaufmann M. Cytokine release from placental endothelial cells, a process associated with preterm labour in the absence of intrauterine infection. Cytokine (1999) 11:66–73. doi: 10.1006/cyto.1998.0399 10080881

[52] Sun B Parks WT Simhan HN Bertolet M Catov JM. Early pregnancy immune profile and preterm birth classified according to uteroplacental lesions. Placenta (2020) 89:99–106. doi: 10.1016/j.placenta.2019.12.007 32056560

[53] Salafia CM Vogel CA Vintzileos AM Bantham KF Pezzullo J Silberman L. Placental pathologic findings in preterm birth. Am J Obstet Gynecol (1991) 165:934–8. doi: 10.1016/0002-9378(91)90443-u 1951558

[54] Kim MJ Romero R Kim CJ Tarca AL Chhauy S LaJeunesse C. Villitis of unknown etiology is associated with a distinct pattern of chemokine up-regulation in the feto-maternal and placental compartments: Implications for conjoint maternal allograft rejection and maternal anti-fetal graft-versus-Host disease. J Immunol (2009) 182:3919–27. doi: 10.4049/jimmunol.0803834 PMC2754231 19265171

[55] Enninga EAL Raber P Quinton RA Ruano R Ikumi N Gray CM. Maternal T cells in the human placental villi support an allograft response during noninfectious villitis. J Immunol Baltim Md 1950 (2020) 204:2931–9. doi: 10.4049/jimmunol.1901297 PMC7307888 32321754

[56] Kim M-A Lee YS Seo K. Assessment of predictive markers for placental inflammatory response in preterm births. PLoS One (2014) 9:e107880. doi: 10.1371/journal.pone.0107880 25291377 PMC4188518

[57] Parris KM Amabebe E Cohen MC Anumba DO. Placental microbial–metabolite profiles and inflammatory mechanisms associated with preterm birth. J Clin Pathol (2020) 74:10–8. doi: 10.1136/jclinpath-2020-206536 32796048

[58] Lappas M Permezel M Rice GE. Mitogen-activated protein kinase proteins regulate LPS-stimulated release of pro-inflammatory cytokines and prostaglandins from human gestational tissues. Placenta (2007) 28:936–45. doi: 10.1016/j.placenta.2007.02.009 17433832

[59] Keelan JA Khan S Yosaatmadja F Mitchell MD. Prevention of inflammatory activation of human gestational membranes in an ex vivo model using a pharmacological NF-κB inhibitor. J Immunol (2009) 183:5270–8. doi: 10.4049/jimmunol.0802660 19783681

[60] Lien Y-C Zhang Z Barila G Green-Brown A Elovitz MA Simmons RA. Intrauterine inflammation alters the transcriptome and metabolome in placenta. Front Physiol (2020) 11:592689. doi: 10.3389/fphys.2020.592689 33250783 PMC7674943

[61] Renaud SJ Sullivan R Graham CH. Tumour necrosis factor alpha stimulates the production of monocyte chemoattractants by extravillous trophoblast cells *via* differential activation of MAPK pathways. Placenta (2009) 30:313–9. doi: 10.1016/j.placenta.2009.01.001 19201463

[62] Cindrova-Davies T Spasic-Boskovic O Jauniaux E Charnock-Jones DS Burton GJ. Nuclear factor-κB, p38, and stress-activated protein kinase mitogen-activated protein kinase signaling pathways regulate proinflammatory cytokines and apoptosis in human placental explants in response to oxidative stress effects of antioxidant vitamins. Am J Pathol (2007) 170:1511–20. doi: 10.2353/ajpath.2007.061035 PMC1854947 17456758

[63] Shirasuna K Seno K Ohtsu A Shiratsuki S Ohkuchi A Suzuki H. AGEs and HMGB1 increase inflammatory cytokine production from human placental cells, resulting in an enhancement of monocyte migration. Am J Reprod Immunol (2016) 75:557–68. doi: 10.1111/aji.12506 26961863

[64] Gómez-Chávez F Correa D Navarrete-Meneses P Cancino-Diaz JC Cancino-Diaz ME Rodríguez-Martínez S. NF-κB and its regulators during pregnancy. Front Immunol (2021) 12:679106. doi: 10.3389/fimmu.2021.679106 34025678 PMC8131829

[65] Stewart PM Whorwood CB Mason JI. Type 2 11β-hydroxysteroid dehydrogenase in foetal and adult life. J Steroid Biochem Mol Biol (1995) 55:465–71. doi: 10.1016/0960-0760(95)00195-6 8547171

[66] Kossintseva I Wong S Johnstone E Guilbert L Olson DM Mitchell BF. Proinflammatory cytokines inhibit human placental 11β-hydroxysteroid dehydrogenase type 2 activity through Ca2+ and cAMP pathways. Am J Physiol-endoc M (2006) 290:E282–8. doi: 10.1152/ajpendo.00328.2005 16174654

[67] Petraglia F Garuti GC Ramundo BD Angioni S Genazzani AR Bilezikjian LM. Mechanism of action of interleukin-1β in increasing corticotropin-releasing factor and adrenocorticotropin hormone release from cultured human placental cells. Am J Obstet Gynecol (1990) 163:1307–12. doi: 10.1016/0002-9378(90)90711-f 2171341

[68] Vitoratos N Mastorakos G Kountouris A Papadias K Creatsas G. Positive association of serum interleukin-1β and CRH levels in women with pre-term labor. J Endocrinol Invest (2007) 30:35–40. doi: 10.1007/bf03347393 17318020

[69] Christiaens I Zaragoza DB Guilbert L Robertson SA Mitchell BF Olson DM. Inflammatory processes in preterm and term parturition. J Reprod Immunol (2008) 79:50–7. doi: 10.1016/j.jri.2008.04.002 18550178

[70] Mitchell MD Dudley DJ Edwin SS Schiller SL. Interleukin-6 stimulates prostaglandin production by human amnion and decidual cells. Eur J Pharmacol (1991) 192:189–91. doi: 10.1016/0014-2999(91)90090-d 2040361

[71] Kniss DA Rovin B Fertel RH Zimmerman PD. Blockade NF-κB activation prohibits TNF-α-induced cyclooxygenase-2 gene expression in ED27Trophoblast-like cells. Placenta (2001) 22:80–9. doi: 10.1053/plac.2000.0591 11162356

[72] Young A Thomson AJ Ledingham M Jordan F Greer IA Norman JE. Immunolocalization of proinflammatory cytokines in myometrium, cervix, and fetal membranes during human parturition at term. Biol Reprod (2002) 66:445–9. doi: 10.1095/biolreprod66.2.445 11804961

[73] Menon R Debnath C Lai A Guanzon D Bhatnagar S Kshetrapal P. Protein profile changes in circulating placental extracellular vesicles in term and preterm births: A longitudinal study. Endocrinology (2020) 161:bqaa009. doi: 10.1210/endocr/bqaa009 31995166 PMC7102872

[74] Driessler F Venstrom K Sabat R Asadullah K Schottelius αAj. Molecular mechanisms of interleukin-10-mediated inhibition of NF-κB activity: a role for p50. Clin Exp Immunol (2004) 135:64–73. doi: 10.1111/j.1365-2249.2004.02342.x 14678266 PMC1808913

[75] Hanna N Bonifacio L Weinberger B Reddy P Murphy S Romero R. Evidence for interleukin-10-Mediated inhibition of cyclo- oxygenase-2 expression and prostaglandin production in preterm human placenta. Am J Reprod Immunol (2006) 55:19–27. doi: 10.1111/j.1600-0897.2005.00342.x 16364008

[76] Robertson SA Skinner RJ Care AS. Essential role for IL-10 in resistance to lipopolysaccharide-induced preterm labor in mice. J Immunol (2006) 177:4888–96. doi: 10.4049/jimmunol.177.7.4888 16982931

[77] Challis Sloboda D Alfaidy N Lye S Gibb W Patel F Whittle W. Prostaglandins and mechanisms of preterm birth. Reproduction (2002) 124:1–17. doi: 10.1530/rep.0.1240001 12090913

[78] Sundrani D Narang A Mehendale S Joshi S Chavan-Gautam P. Investigating the expression of MMPs and TIMPs in preterm placenta and role of CpG methylation in regulating MMP-9 expression. IUBMB Life (2017) 69:985–93. doi: 10.1002/iub.1687 29130646

[79] Sundrani DP Chavan-Gautam PM Pisal HR Mehendale SS Joshi SR. Matrix metalloproteinase-1 and -9 in human placenta during spontaneous vaginal delivery and caesarean sectioning in preterm pregnancy. PloS One (2012) 7:e29855. doi: 10.1371/journal.pone.0029855 22253805 PMC3257231

[80] Mitchell JA Lye SJ. Differential expression of activator protein-1 transcription factors in pregnant rat Myometrium1. Biol Reprod (2002) 67:240–6. doi: 10.1095/biolreprod67.1.240 12080023

[81] Hirota Y Daikoku T Tranguch S Xie H Bradshaw HB Dey SK. Uterine-specific p53 deficiency confers premature uterine senescence and promotes preterm birth in mice. J Clin Invest (2010) 120:803–15. doi: 10.1172/jci40051 PMC2827950 20124728

[82] Moawad AH Goldenberg RL Mercer B Meis PJ Iams JD Das A. The preterm prediction study: The value of serum alkaline phosphatase, α-fetoprotein, plasma corticotropin-releasing hormone, and other serum markers for the prediction of spontaneous preterm birth. Am J Obstet Gynecol (2002) 186:990–6. doi: 10.1067/mob.2002.121727 12015526

[83] Ellis MJ Livesey JH Inder WJ Prickett TCR Reid R. Plasma corticotropin-releasing hormone and unconjugated estriol in human pregnancy: Gestational patterns and ability to predict preterm delivery. Am J Obstet Gynecol (2002) 186:94–9. doi: 10.1067/mob.2002.119188 11810092

[84] Wadhwa PD Garite TJ Porto M Glynn L Chicz-DeMet A Dunkel-Schetter C. Placental corticotropin-releasing hormone (CRH), spontaneous preterm birth, and fetal growth restriction: A prospective investigation. Am J Obstet Gynecol (2004) 191:1063–9. doi: 10.1016/j.ajog.2004.06.070 15507922

[85] Stamatelou F Deligeoroglou E Farmakides G Creatsas G. Abnormal progesterone and corticotropin releasing hormone levels are associated with preterm labour. Ann Acad Med Singap (2009) 1–16. doi: 10.47102/annals-acadmedsg.V38N11p1011 19956826

[86] Herrera CL Bowman ME McIntire DD Nelson DB Smith R. Revisiting the placental clock: Early corticotrophin-releasing hormone rise in recurrent preterm birth. PLoS One (2021) 16:e0257422. doi: 10.1371/journal.pone.0257422 34529698 PMC8445461

[87] Gross G Imamura T Vogt SK Wozniak DF Nelson DM Sadovsky Y. Inhibition of cyclooxygenase-2 prevents inflammation-mediated preterm labor in the mouse. Am J Physiol-Regul Integr Comp Physiol (2000) 278:R1415–23. doi: 10.1152/ajpregu.2000.278.6.r1415 10848506

[88] Kishore AH Liang H Kanchwala M Xing C Ganesh T Akgul Y. Prostaglandin dehydrogenase is a target for successful induction of cervical ripening. Proc Natl Acad Sci (2017) 114:E6427–36. doi: 10.1073/pnas.1704945114 PMC5547630 28716915

[89] Kramer MS Lydon J Séguin L Goulet L Kahn SR McNamara H. Stress pathways to spontaneous preterm birth: The role of stressors, psychological distress, and stress hormones. Am J Epidemiol (2009) 169:1319–26. doi: 10.1093/aje/kwp061 19363098

[90] Zachariades E Mparmpakas D Pang Y Rand-Weaver M Thomas P Karteris E. Changes in placental progesterone receptors in term and preterm labour. Placenta (2012) 33:367–72. doi: 10.1016/j.placenta.2012.01.002 22341631

[91] Langmia IM Apalasamy YD Omar SZ Mohamed Z. Progesterone receptor (PGR) gene polymorphism is associated with susceptibility to preterm birth. BMC Med Genet (2015) 16:63. doi: 10.1186/s12881-015-0202-1 26286601 PMC4593226

[92] Diaz-Cueto L Dominguez-Lopez P Cantillo-Cabarcas J Perez-Figueroa G Arechavaleta-Velasco M Arechavaleta-Velasco F. Progesterone receptor gene polymorphisms are not associated with preterm birth in a Hispanic population. Int J Gynecol Obstet (2008) 103:153–7. doi: 10.1016/j.ijgo.2008.06.008 18722616

[93] Morgan TK. Placental insufficiency is a leading cause of preterm labor. Neoreviews (2014) 15:e518–25. doi: 10.1542/neo.15-12-e518

[94] Lekovich J Stewart J Anderson S Niemasik E Pereira N Chasen S. Placental malperfusion as a possible mechanism of preterm birth in patients with müllerian anomalies. J Perinat Med (2017) 45:45–9. doi: 10.1515/jpm-2016-0075 27639266

[95] Baumfeld Y Herskovitz R Niv ZB Mastrolia SA Weintraub AY. Placenta associated pregnancy complications in pregnancies complicated with placenta previa. Taiwan J Obstetrics Gynecol (2017) 56:331–5. doi: 10.1016/j.tjog.2017.04.012 28600043

[96] Erez O Novack L Klaitman V Erez-Weiss I Beer-Weisel R Dukler D. Early preterm delivery due to placenta previa is an independent risk factor for a subsequent spontaneous preterm birth. BMC Pregnancy Childb (2012) 12:82–2. doi: 10.1186/1471-2393-12-82 PMC3489587 22876799

[97] Hua M Odibo AO Longman RE Macones GA Roehl KA Cahill AG. Congenital uterine anomalies and adverse pregnancy outcomes. Am J Obstet Gynecol (2011) 205:558.e1–5. doi: 10.1016/j.ajog.2011.07.022 21907963

[98] Delforce SJ Lumbers ER Ellery SJ Murthi P Pringle K. Dysregulation of the placental renin–angiotensin system in human fetal growth restriction. Reproduction (2019) 1:237–45. doi: 10.1530/rep-18-0633 31247590

[99] Yart L Bahmanyar ER Cohen M Tejada BMDE. Role of the uteroplacental renin–angiotensin system in placental development and function, and its implication in the preeclampsia pathogenesis. Biomed (2021) 9:1332. doi: 10.3390/biomedicines9101332 PMC8533592 34680449

[100] Tertemiz F Asar M Demir R Cakmak H Arici A Kayisli UA. Phospho-akt and PTEN expression in normal and preeclamptic placentas: Possible role for phospho-akt in endothelial dysfunction and reduced trophoblast invasion in preeclampsia. Fertil Steril (2005) 84:S111. doi: 10.1016/j.fertnstert.2005.07.269

[101] Morgan T. Role of the placenta in preterm birth: A review. Am J Perinat (2016) 33:258–66. doi: 10.1055/s-0035-1570379 26731184

[102] Xue P Fan W Diao Z Li Y Kong C Dai X. Up-regulation of PTEN *via* LPS/AP-1/NF-κB pathway inhibits trophoblast invasion contributing to preeclampsia. Mol Immunol (2020) 118:182–90. doi: 10.1016/j.molimm.2019.12.018 31896494

[103] Kramer BW Kaemmerer U Kapp M Herbst D Marx A Berg D. Decreased expression of angiogenic factors in placentas with chorioamnionitis after preterm birth. Pediatr Res (2005) 58:607–12. doi: 10.1203/01.pdr.0000175641.39056.7a 16148081

[104] Goland R Tropper P Warren W Stark R Jozak S Conwell I. Concentrations of corticotrophin-releasing hormone in the umbilical-cord blood of pregnancies complicated by pre-eclampsia. Reprod Fertility Dev (1995) 7:1227–30. doi: 10.1071/rd9951227 8848592

[105] Menon R. Human fetal membranes at term: Dead tissue or signalers of parturition? Placenta (2016) 44:1–5. doi: 10.1016/j.placenta.2016.05.013 27452431 PMC5375105

[106] Menon R Richardson LS Lappas M. Fetal membrane architecture, aging and inflammation in pregnancy and parturition. Placenta (2018) 79:40–5. doi: 10.1016/j.placenta.2018.11.003 PMC7041999 30454905

[107] Menon R Moore JJ. Fetal membranes, not a mere appendage of the placenta, but a critical part of the fetal-maternal interface controlling parturition. Obstet Gyn Clin N Am (2019) 47:147–62. doi: 10.1016/j.ogc.2019.10.004 32008665

[108] Polettini J Behnia F Taylor BD Saade GR Taylor RN Menon R. Telomere fragment induced amnion cell senescence: A contributor to parturition? PloS One (2015) 10:e0137188. doi: 10.1371/journal.pone.0137188 26397719 PMC4580414

[109] Hay ED. Extracellular matrix. J Cell Biol (1981) 91:205s–23s. doi: 10.1083/jcb.91.3.205s PMC2112832 6172429

[110] Ilancheran S Moodley Y Manuelpillai U. Human fetal membranes: A source of stem cells for tissue regeneration and repair? Placenta (2009) 30:2–10. doi: 10.1016/j.placenta.2008.09.009 18995896

[111] Bryant-Greenwood GD. The extracellular matrix of the human fetal membranes: Structure and function. Placenta (1998) 19:1–11. doi: 10.1016/s0143-4004(98)90092-3 9481779

[112] Strauss JF. Extracellular matrix dynamics and fetal membrane rupture. Reprod Sci (2013) 20:140–53. doi: 10.1177/1933719111424454 PMC3826277 22267536

[113] Kaufmann P Mühlhauser J Crescimanno C Castellucci M Rajaniemi H Parkkila S. Immunohistochemistry of carbonic anhydrase in human placenta and fetal membranes. Histochemistry (1994) 101:91–8. doi: 10.1007/bf00269354 8071088

[114] Mamede AC Carvalho MJ Abrantes AM Laranjo M Maia CJ Botelho MF. Amniotic membrane: from structure and functions to clinical applications. Cell Tissue Res (2012) 349:447–58. doi: 10.1007/s00441-012-1424-6 22592624

[115] Takahashi N Okuno T Fujii H Makino S Takahashi M Ohba M. Up-regulation of cytosolic prostaglandin e synthase in fetal-membrane and amniotic prostaglandin E2 accumulation in labor. PloS One (2021) 16:e0250638. doi: 10.1371/journal.pone.0250638 33891661 PMC8064594

[116] Alfaidy N Sun M Challis JRG Gibb W. Expression of membrane prostaglandin e synthase in human placenta and fetal membranes and effect of labor. Endocrine (2003) 20:219–25. doi: 10.1385/endo:20:3:219 12721500

[117] Duchesne MJ Thaler-Dao H Paulet ACDE. Prostaglandin synthesis in human placenta and fetal membranes. Prostaglandins (1978) 15:19–42. doi: 10.1016/s0090-6980(78)80003-3 24235

[118] Whittle WL Gibb W Challis JRG. The characterization of human amnion epithelial and mesenchymal cells: the cellular expression, activity and glucocorticoid regulation of prostaglandin output. Placenta (2000) 21:394–401. doi: 10.1053/plac.1999.0482 10833375

[119] Nakla S Skinner K Mitchell BF Challis JRG. Changes in prostaglandin transfer across human fetal membranes obtained after spontaneous labor. Am J Obstet Gynecol (1986) 155:1337–41. doi: 10.1016/0002-9378(86)90170-5 3466546

[120] Bennett PR Chamberlain GVP Patel L Elder MG Myatt L. Mechanisms of parturition: The transfer of prostaglandin E2 and 5-hydroxyeicosatetraenoic acid across fetal membranes. Am J Obstet Gynecol (1990) 162:683–7. doi: 10.1016/0002-9378(90)90984-f 2316568

[121] Mitchell BF Rogers K Wong S. The dynamics of prostaglandin metabolism in human fetal membranes and decidua around the time of parturition. J Clin Endocrinol Metab (1993) 77:759–64. doi: 10.1210/jcem.77.3.8370697 8370697

[122] McLaren J Malak TM Bell SC. Structural characteristics of term human fetal membranes prior to labour: identification of an area of altered morphology overlying the cervix. Hum Reprod (1999) 14:237–41. doi: 10.1093/humrep/14.1.237 10374127

[123] Maymon E Romero R Pacora P Gervasi M-T Bianco K Ghezzi F. Evidence for the participation of interstitial collagenase (matrix metalloproteinase 1) in preterm premature rupture of membranes. Am J Obstet Gynecol (2000) 183:914–20. doi: 10.1067/mob.2000.108879 11035337

[124] Khwad ME Pandey V Stetzer B Mercer BM Kumar D Moore RM. Fetal membranes from term VAginal deliveries have a zone of weakness exhibiting characteristics of apoptosis and remodeling. J Soc Gynecol Invest (2006) 13:191–5. doi: 10.1016/j.jsgi.2005.12.010 16638590

[125] Strohl A Kumar D Novince R Shaniuk P Smith J Bryant K. Decreased adherence and spontaneous separation of fetal membrane layers–amnion and choriodecidua–a possible part of the normal weakening process. Placenta (2009) 31:18–24. doi: 10.1016/j.placenta.2009.10.012 19922998 PMC2808440

[126] Murphy BEP. Chorionic membrane as an extra-adrenal source of foetal cortisol in human amniotic fluid. Nature (1977) 266:179–81. doi: 10.1038/266179a0 859592

[127] Murphy BEP. Ontogeny of cortisol-cortisone interconversion in human tissues: A role for cortisone in human fetal development. J Steroid Biochemist (1981) 14:811–7. doi: 10.1016/0022-4731(81)90226-0 6946266

[128] Sun K Yang K Challis JR. Differential expression of 11 beta-hydroxysteroid dehydrogenase types 1 and 2 in human placenta and fetal membranes. J Clin Endocrinol Metab (1997) 82:300–5. doi: 10.1210/jcem.82.1.3681 8989277

[129] Wang W-S Guo C-M Sun K. Cortisol regeneration in the fetal membranes, a coincidental or requisite event in human parturition? Front Physiol (2020) 11:462. doi: 10.3389/fphys.2020.00462 32523541 PMC7261858

[130] Liu C Guo C Wang W Zhu P Li W Mi Y. Inhibition of lysyl oxidase by cortisol regeneration in human amnion: Implications for rupture of fetal membranes. Endocrinology (2016) 157:4055–65. doi: 10.1210/en.2016-1406 27533889

[131] Alfaidy N Li W MacIntosh T Yang K Challis J. Late gestation increase in 11beta-hydroxysteroid dehydrogenase 1 expression in human fetal membranes: a novel intrauterine source of cortisol. J Clin Endocrinol Metab (2003) 88:5033–8. doi: 10.1210/jc.2002-021915 14557491

[132] Mosaad E Peiris HN Holland O Garcia IM Mitchell MD. The role(s) of eicosanoids and exosomes in human parturition. Front Physiol (2020) 11:594313. doi: 10.3389/fphys.2020.594313 33424622 PMC7786405

[133] Menon R Boldogh I Hawkins HK Woodson M Polettini J Syed TA. Histological evidence of oxidative stress and premature senescence in preterm premature rupture of the human fetal membranes recapitulated in vitro. Am J Pathol (2014) 184:1740–51. doi: 10.1016/j.ajpath.2014.02.011 24832021

[134] Behnia F Taylor BD Woodson M Kacerovsky M Hawkins H Fortunato SJ. Chorioamniotic membrane senescence: a signal for parturition? Am J Obstet Gynecol (2015) 213:359.e1–359.e16. doi: 10.1016/j.ajog.2015.05.041 26025293

[135] Menon R Behnia F Polettini J Saade GR Campisi J Velarde M. Placental membrane aging and HMGB1 signaling associated with human parturition. Aging Albany Ny (2016) 8:216–29. doi: 10.18632/aging.100891 PMC4789578 26851389

[136] Menon R Boldogh I Urrabaz-Garza R Polettini J Syed TA Saade GR. Senescence of primary amniotic cells *via* oxidative DNA damage. PloS One (2013) 8:e83416. doi: 10.1371/journal.pone.0083416 24386195 PMC3873937

[137] Roh JS Sohn DH. Damage-associated molecular patterns in inflammatory diseases. Immune Netw (2018) 18:e27. doi: 10.4110/in.2018.18.e27 30181915 PMC6117512

[138] Bredeson S Papaconstantinou J Deford JH Kechichian T Syed TA Saade GR. HMGB1 promotes a p38MAPK associated non-infectious inflammatory response pathway in human fetal membranes. PloS One (2014) 9:e113799. doi: 10.1371/journal.pone.0113799 25469638 PMC4254744

[139] Menon R Mesiano S Taylor RN. Programmed fetal membrane senescence and exosome-mediated signaling: A mechanism associated with timing of human parturition. Front Endocrinol (2017) 8:196. doi: 10.3389/fendo.2017.00196 PMC5562683 28861041

[140] Sheller-Miller S Trivedi J Yellon SM Menon R. Exosomes cause preterm birth in mice: Evidence for paracrine signaling in pregnancy. Sci Rep-uk (2019) 9:608. doi: 10.1038/s41598-018-37002-x PMC6345869 30679631

[141] Jin J Menon R. Placental exosomes: A proxy to understand pregnancy complications. Am J Reprod Immunol (2018) 79:e12788. doi: 10.1111/aji.12788 29193463 PMC5908735

[142] Sheller S Papaconstantinou J Urrabaz-Garza R Richardson L Saade G Salomon C. Amnion-Epithelial-Cell-Derived exosomes demonstrate physiologic state of cell under oxidative stress. PLoS One (2016) 11:e0157614. doi: 10.1371/journal.pone.0157614 27333275 PMC4917104

[143] Shahin HI Radnaa E Tantengco OAG Kechichian T Kammala AK Sheller-Miller S. Microvesicles and exosomes released by amnion epithelial cells under oxidative stress cause inflammatory changes in uterine cells. Biol Reprod (2021) 105:464–80. doi: 10.1093/biolre/ioab088 PMC8335356 33962471

[144] Hadley EE Sheller-Miller S Saade G Salomon C Mesiano S Taylor RN. Amnion epithelial cell–derived exosomes induce inflammatory changes in uterine cells. Am J Obstet Gynecol (2018) 219:478.e1–478.e21. doi: 10.1016/j.ajog.2018.08.021 30138617 PMC6239974

[145] Sheller-Miller S Urrabaz-Garza R Saade G Menon R. Damage-associated molecular pattern markers HMGB1 and cell-free fetal telomere fragments in oxidative-stressed amnion epithelial cell-derived exosomes. J Reprod Immunol (2017) 123:3–11. doi: 10.1016/j.jri.2017.08.003 28858636 PMC5632595

[146] Chang G Mouillet J Mishima T Chu T Sadovsky E Coyne CB. Expression and trafficking of placental microRNAs at the feto-maternal interface. FASEB J (2017) 31:2760–70. doi: 10.1096/fj.201601146r PMC5471515 28289056

[147] Sheller-Miller S Lei J Saade G Salomon C Burd I Menon R. Feto-maternal trafficking of exosomes in murine pregnancy models. Front Pharmacol (2016) 7:432. doi: 10.3389/fphar.2016.00432 27895585 PMC5108780

[148] Richardson LS Vargas G Brown T Ochoa L Sheller-Miller S Saade GR. Discovery and characterization of human amniochorionic membrane microfractures. Am J Pathol (2017) 187:2821–30. doi: 10.1016/j.ajpath.2017.08.019 PMC5718093 28939208

[149] Richardson L Vargas G Brown T Ochoa L Trivedi J Kacerovský M. Redefining 3Dimensional placental membrane microarchitecture using multiphoton microscopy and optical clearing. Placenta (2017) 53:66–75. doi: 10.1016/j.placenta.2017.03.017 28487023

[150] Richardson L Menon R. Proliferative, migratory, and transition properties reveal metastate of human amnion cells. Am J Pathol (2018) 188:2004–15. doi: 10.1016/j.ajpath.2018.05.019 PMC6119821 29981743

[151] Behnia F Sheller S Menon R. Mechanistic differences leading to infectious and sterile inflammation. Am J Reprod Immunol (2016) 75:505–18. doi: 10.1111/aji.12496 26840942

[152] Silva M de C Richardson LS Kechichian T Urrabaz-Garza R Silva MGDA Menon R. Inflammation, but not infection, induces EMT in human amnion epithelial cells. Reproduction (2020) 160:627–38. doi: 10.1530/rep-20-0283 32841157

[153] Richardson LS Taylor RN Menon R. Reversible EMT and MET mediate amnion remodeling during pregnancy and labor. Sci Signal (2020) 13, 1–16. doi: 10.1126/scisignal.aay1486 PMC7092701 32047115

[154] Menon R Behnia F Polettini J Richardson LS. Novel pathways of inflammation in human fetal membranes associated with preterm birth and preterm pre-labor rupture of the membranes. Semin Immunopathol (2020) 42:431–50. doi: 10.1007/s00281-020-00808-x PMC9296260 32785751

[155] Jin J Richardson L Sheller-Miller S Zhong N Menon R. Oxidative stress induces p38MAPK-dependent senescence in the feto-maternal interface cells. Placenta (2018) 67:15–23. doi: 10.1016/j.placenta.2018.05.008 29941169 PMC6023622

[156] Polettini J Richardson LS Menon R. Oxidative stress induces senescence and sterile inflammation in murine amniotic cavity. Placenta (2018) 63:26–31. doi: 10.1016/j.placenta.2018.01.009 29486853 PMC5833301

[157] Dixon CL Richardson L Sheller-Miller S Saade G Menon R. A distinct mechanism of senescence activation in amnion epithelial cells by infection, inflammation, and oxidative stress. Am J Reprod Immunol (2018) 79:e12790. doi: 10.1111/aji.12790 PMC5815890 29193446

[158] Finkel T. Oxidant signals and oxidative stress. Curr Opin Cell Biol (2003) 15:247–54. doi: 10.1016/s0955-0674(03)00002-4 12648682

[159] Hewitt G Jurk D Marques FDM Correia-Melo C Hardy T Gackowska A. Telomeres are favoured targets of a persistent DNA damage response in ageing and stress-induced senescence. Nat Commun (2012) 3:708. doi: 10.1038/ncomms1708 22426229 PMC3292717

[160] Richardson L Dixon CL Aguilera-Aguirre L Menon R. Oxidative stress-induced TGF-beta/TAB1-mediated p38MAPK activation in human amnion epithelial cells. Biol Reprod (2018) 99:1100–12. doi: 10.1093/biolre/ioy135 PMC7190655 29893818

[161] King AE Kelly RW Sallenave J-M Bocking AD Challis JRG. Innate immune defences in the human uterus during pregnancy. Placenta (2007) 28:1099–106. doi: 10.1016/j.placenta.2007.06.002 17664005

[162] Stock SJ Kelly RW Riley SC Calder AA. Natural antimicrobial production by the amnion. Am J Obstet Gynecol (2007) 196:255.e1–6. doi: 10.1016/j.ajog.2006.10.908 17346544

[163] Kim CJ Romero R Chaemsaithong P Chaiyasit N Yoon BH Kim YM. Acute chorioamnionitis and funisitis: definition, pathologic features, and clinical significance. Am J Obstet Gynecol (2015) 213:S29–52. doi: 10.1016/j.ajog.2015.08.040 PMC4774647 26428501

[164] Romero R Chaemsaithong P Korzeniewski SJ Tarca AL Bhatti G Xu Z. Clinical chorioamnionitis at term II: the intra-amniotic inflammatory response. J Perinat Med (2016) 44:5–22. doi: 10.1515/jpm-2015-0045 25938217 PMC5891100

[165] Gomez-Lopez N Romero R Galaz J Xu Y Panaitescu B Slutsky R. Cellular immune responses in amniotic fluid of women with preterm labor and intra-amniotic infection or intra-amniotic inflammation. Am J Reprod Immunol (2019) 82:e13171. doi: 10.1111/aji.13171 31323170 PMC6788958

[166] Ramuta αTŽ Šket T Erjavec MS Kreft ME. Antimicrobial activity of human fetal membranes: From biological function to clinical use. Front Bioeng Biotechnol (2021) 9:691522. doi: 10.3389/fbioe.2021.691522 34136474 PMC8201995

[167] Redline RW. Inflammatory response in acute chorioamnionitis. Semin Fetal Neonatal Med (2012) 17:20–5. doi: 10.1016/j.siny.2011.08.003 21865101

[168] Presicce P Park C-W Senthamaraikannan P Bhattacharyya S Jackson C Kong F. IL-1 signaling mediates intrauterine inflammation and chorio-decidua neutrophil recruitment and activation. JCI Insight (2018) 3:e98306. doi: 10.1172/jci.insight.98306 PMC5926925 29563340

[169] Martinez-Varea A Romero R Xu Y Miller D Ahmed AI Chaemsaithong P. Clinical chorioamnionitis at term VII: the amniotic fluid cellular immune response. J Perinat Med (2017) 45:523–38. doi: 10.1515/jpm-2016-0225 PMC5624709 27763883

[170] Presicce P Senthamaraikannan P Alvarez M Rueda CM Cappelletti M Miller LA. Neutrophil recruitment and activation in decidua with intra-amniotic IL-1beta in the preterm rhesus macaque. Biol Reprod (2015) 92:56. doi: 10.1095/biolreprod.114.124420 25537373 PMC4342792

[171] Dudley DJ Edwin SS Dangerfield A Jackson K Trautman MS. Regulation of decidual cell and chorion cell production of interleukin-10 by purified bacterial products. Am J Reprod Immunol (1997) 38:246–51. doi: 10.1111/j.1600-0897.1997.tb00510.x 9352010

[172] Athayde N Romero R Gomez R Maymon E Pacora P Mazor M. Matrix metalloproteinases-9 in preterm and term human parturition. J Maternal-fetal Neonatal Med (1999) 8:213–9. doi: 10.3109/14767059909052049 10475503

[173] Vadillo-Ortega F González-Avila G Furth EE Lei H Muschel RJ Stetler-Stevenson WG. 92-kd type IV collagenase (matrix metalloproteinase-9) activity in human amniochorion increases with labor. Am J Pathol (1995) 146:148–56. PMC1870762 7856724

[174] Maymon E Romero R Pacora P Gomez R Athayde N Edwin S. Human neutrophil collagenase (matrix metalloproteinase 8) in parturition, premature rupture of the membranes, and intrauterine infection. Am J Obstet Gynecol (2000) 183:94–9. doi: 10.1067/mob.2000.105344 10920315

[175] Helmig BR Romero R Espinoza J Chaiworapongsa T Bujold E Gomez R. Neutrophil elastase and secretory leukocyte protease inhibitor in prelabor rupture of membranes, parturition and intra-amniotic infection. J Maternal-fetal Neonatal Med (2009) 12:237–46. doi: 10.1080/jmf.12.4.237.246 12572592

[176] DiGiulio DB Romero R Kusanovic JP Gómez R Kim CJ Seok KS. Prevalence and diversity of microbes in the amniotic fluid, the fetal inflammatory response, and pregnancy outcome in women with preterm pre-labor rupture of membranes. Am J Reprod Immunol (2010) 64:38–57. doi: 10.1111/j.1600-0897.2010.00830.x 20331587 PMC2907911

[177] Arechavaleta-Velasco F Ogando D Parry S Vadillo-Ortega F. Production of matrix metalloproteinase-9 in lipopolysaccharide-stimulated human amnion occurs through an autocrine and paracrine proinflammatory cytokine-dependent system. Biol Reprod (2002) 67:1952–8. doi: 10.1095/biolreprod.102.004721 12444074

[178] Fortunato SJ Menon R. IL-1 beta is a better inducer of apoptosis in human fetal membranes than IL-6. Placenta (2003) 24:922–8. doi: 10.1016/s0143-4004(03)00160-7 14580374

[179] Menon R Richardson LS. Preterm prelabor rupture of the membranes: A disease of the fetal membranes. Semin Perinatol (2017) 41:409–19. doi: 10.1053/j.semperi.2017.07.012 PMC5659934 28807394

[180] Huang W-C Sala-Newby GB Susana A Johnson JL Newby AC. Classical macrophage activation up-regulates several matrix metalloproteinases through mitogen activated protein kinases and nuclear factor-κB. PloS One (2012) 7:e42507. doi: 10.1371/journal.pone.0042507 22880008 PMC3411745

[181] Pavlov O Pavlova O Ailamazyan E Selkov S. Original article: Characterization of cytokine production by human term placenta macrophages *In vitro*: Characterization of cytokine production by human term placenta macrophages. Am J Reprod Immunol (2008) 60:556–67. doi: 10.1111/j.1600-0897.2008.00657.x 18853988

[182] Stygar D Wang H Vladic YS Ekman G Eriksson H Sahlin L. Increased level of matrix metalloproteinases 2 and 9 in the ripening process of the human cervix. Biol Reprod (2002) 67:889–94. doi: 10.1095/biolreprod.102.005116 12193399

[183] Menzies FM Shepherd MC Nibbs RJ Nelson SM. The role of mast cells and their mediators in reproduction, pregnancy and labour. Hum Reprod Update (2011) 17:383–96. doi: 10.1093/humupd/dmq053 20959350

[184] Garfield RE Bytautiene E Vedernikov YP Marshall JS Romero R. Modulation of rat uterine contractility by mast cells and their mediators. Am J Obstet Gynecol (2000) 183:118–25. doi: 10.1067/mob.2000.105741 10920318

[185] Juremalm M Nilsson G. Chemokine receptor expression by mast cells. Chem Immunol Allergy (2005) 87:130–44. doi: 10.1159/000087640 16107768

[186] Törnblom SA Klimaviciute A Byström B Chromek M Brauner A Ekman-Ordeberg G. Non-infected preterm parturition is related to increased concentrations of IL-6, IL-8 and MCP-1 in human cervix. Reprod Biol Endocrinol Rb E (2005) 3:39–9. doi: 10.1186/1477-7827-3-39 PMC1201172 16122384

[187] Gonzalez JM Franzke C-W Yang F Romero R Girardi G. Complement activation triggers metalloproteinases release inducing cervical remodeling and preterm birth in mice. Am J Pathol (2011) 179:838–49. doi: 10.1016/j.ajpath.2011.04.024 PMC3157168 21801872

[188] Lee J Romero R Xu Y Kim J-S Topping V Yoo W. A signature of maternal anti-fetal rejection in spontaneous preterm birth: Chronic chorioamnionitis, anti-human leukocyte antigen antibodies, and C4d. PLoS One (2011) 6:e16806. doi: 10.1371/journal.pone.0016806 21326865 PMC3033909

[189] Many A Hill LM Lazebnik N Martin JG. The association between polyhydramnios and preterm delivery. Obstet Gynecol (1995) 86:389–91. doi: 10.1016/0029-7844(95)00179-u 7651648

[190] Asadi N Khalili A Zarei Z Azimi A Kasraeian M Foroughinia L. Perinatal outcome in pregnancy with polyhydramnios in comparison with normal pregnancy in department of obstetrics at Shiraz university of medical sciences. J Maternal-fetal Neonatal Med (2017) 31:1–18. doi: 10.1080/14767058.2017.1325864 28462606

[191] Kurdi AM Mesleh RA Al-Hakeem MM Khashoggi TY Khalifa HM. Multiple pregnancy and preterm labor. Saudi Med J (2004) 25:632–7. 15138532

[192] Richardson LS Radnaa E Urrabaz-Garza R Lavu N Menon R. Stretch, scratch, and stress: Suppressors and supporters of senescence in human fetal membranes. Placenta (2020) 99:27–34. doi: 10.1016/j.placenta.2020.07.013 32750642 PMC7530028

[193] Maradny EE Kanayama N Halim A Maehara K Terao T. Stretching of fetal membranes increases the concentration of interleukin-8 and collagenase activity. Am J Obstet Gynecol (1996) 174:843–9. doi: 10.1016/s0002-9378(96)70311-3 8633654

[194] Leguizamón G Smith J Younis H Nelson DM Sadovsky Y. Enhancement of amniotic cyclooxygenase type 2 activity in women with preterm delivery associated with twinsor polyhydramnios. Am J Obstet Gynecol (2001) 184:117–22. doi: 10.1067/mob.2001.108076 11174490

[195] Mohan AR Sooranna SR Lindstrom TM Johnson MR Bennett PR. The effect of mechanical stretch on cyclooxygenase type 2 expression and activator protein-1 and nuclear factor-κB activity in human amnion cells. Endocrinology (2007) 148:1850–7. doi: 10.1210/en.2006-1289 17218407

[196] Manabe Y Yoshimura S Mori T Aso T. Plasma levels of 13,14-dihydro-15-keto prostaglandin F2 alpha, estrogens, and progesterone during stretch-induced labor at term. Prostaglandins (1985) 30:141–52. doi: 10.1016/s0090-6980(85)80018-6 4048477

[197] Kendal-Wright CE. Stretching, mechanotransduction, and proinflammatory cytokines in the fetal membranes. Reprod Sci (2007) 14:35–41. doi: 10.1177/1933719107310763 18089608

[198] Bhunia S O’Brien S Ling Y Huang Z Wu P Yang Y. New approaches suggest term and preterm human fetal membranes may have distinct biomechanical properties. Sci Rep-uk (2022) 12:5109. doi: 10.1038/s41598-022-09005-2 PMC8948223 35332209

[199] Mori M Bogdan A Balassa T Csabai T Szekeres-Bartho J. The decidua–the maternal bed embracing the embryo–maintains the pregnancy. Semin Immunopathol (2016) 38:635–49. doi: 10.1007/s00281-016-0574-0 PMC5065593 27287066

[200] Ander SE Diamond MS Coyne CB. Immune responses at the maternal-fetal interface. Sci Immunol (2019) 4. doi: 10.1126/sciimmunol.aat6114 PMC6744611 30635356

[201] Okada H Tsuzuki T Murata H. Decidualization of the human endometrium. Reprod Med Biol (2018) 17:220–7. doi: 10.1002/rmb2.12088 PMC6046526 30013421

[202] Olson DM. The role of prostaglandins in the initiation of parturition. Best Pract Res Cl Ob (2003) 17:717–30. doi: 10.1016/s1521-6934(03)00069-5 12972010

[203] Hirota Y Cha J Yoshie M Daikoku T Dey SK. Heightened uterine mammalian target of rapamycin complex 1 (mTORC1) signaling provokes preterm birth in mice. P Natl Acad Sci U S A (2011) 108:18073–8. doi: 10.1073/pnas.1108180108 PMC3207648 22025690

[204] Roizen JD Asada M Tong M Tai H-H Muglia LJ. Preterm birth without progesterone withdrawal in 15-hydroxyprostaglandin dehydrogenase hypomorphic mice. Mol Endocrinol (2008) 22:105–12. doi: 10.1210/me.2007-0178 PMC2194629 17872381

[205] Gomez-Lopez N Vega-Sanchez R Castillo-Castrejon M Romero R Cubeiro-Arreola K Vadillo-Ortega F. Evidence for a role for the adaptive immune response in human term parturition. Am J Reprod Immunol New York N Y 1989 (2013) 69:212–30. doi: 10.1111/aji.12074 PMC3600361 23347265

[206] Hamilton S Oomomian Y Stephen G Shynlova O Tower CL Garrod A. Macrophages infiltrate the human and rat decidua during term and preterm labor: evidence that decidual inflammation precedes labor. Biol Reprod (2012) 86:39. doi: 10.1095/biolreprod.111.095505 22011391

[207] Fuchs A-R. Plasma membrane receptors regulating myometrial contractility and their hormonal modulation. Semin Perinatol (1995) 19:15–30. doi: 10.1016/s0146-0005(95)80044-1 7754408

[208] Norwitz ER Robinson JN Challis JRG. The control of labor. N Engl J Med (1999) 341:660–6. doi: 10.1056/nejm199908263410906 10460818

[209] Challis JRG Matthews SG Gibb W Lye SJ. Endocrine and paracrine regulation of birth at term and preterm. Endocr Rev (2000) 21:514–50. doi: 10.1210/edrv.21.5.0407 11041447

[210] Kota SK Gayatri K Jammula S Kota SK Krishna SVS Meher LK. Endocrinology of parturition. Indian J Endocrinol Metab (2013) 17:50–9. doi: 10.4103/2230-8210.107841 PMC3659907 23776853

[211] Moore RM Mansour JM Redline RW Mercer BM Moore JJ. The physiology of fetal membrane rupture: Insight gained from the determination of physical properties. Placenta (2006) 27:1037–51. doi: 10.1016/j.placenta.2006.01.002 16516962

[212] Manuck TA Esplin MS Biggio J Bukowski R Parry S Zhang H. The phenotype of spontaneous preterm birth: application of a clinical phenotyping tool. Am J Obstet Gynecol (2015) 212:487.e1–487.e11. doi: 10.1016/j.ajog.2015.02.010 25687564 PMC4456184

[213] Deng W Yuan J Cha J Sun X Bartos A Yagita H. Endothelial cells in the decidual bed are potential therapeutic targets for preterm birth prevention. Cell Rep (2019) 27:1755–68.e4. doi: 10.1016/j.celrep.2019.04.049 31067461 PMC6554729

[214] Xu Y Romero R Miller D Kadam L Mial TN Plazyo O. An M1-like macrophage polarization in decidual tissue during spontaneous preterm labor that is attenuated by rosiglitazone treatment. J Immunol (2016) 196:2476–91. doi: 10.4049/jimmunol.1502055 PMC4779725 26889045

[215] Presicce P Cappelletti M Senthamaraikannan P Ma F Morselli M Jackson CM. TNF-signaling modulates neutrophil-mediated immunity at the feto-maternal interface during LPS-induced intrauterine inflammation. Front Immunol (2020) 11:558. doi: 10.3389/fimmu.2020.00558 32308656 PMC7145904

[216] Flores-Herrera H García-López G Díaz NF Molina-Hernández A Osorio-Caballero M Soriano-Becerril D. An experimental mixed bacterial infection induced differential secretion of proinflammatory cytokines (IL-1β, TNFα) and proMMP-9 in human fetal membranes. Placenta (2012) 33:271–7. doi: 10.1016/j.placenta.2012.01.007 22280559

[217] Lockwood CJ Arcuri F Toti P Felice CD Krikun G Guller S. Tumor necrosis factor-α and interleukin-1β regulate interleukin-8 expression in third trimester decidual cells. Am J Pathol (2006) 169:1294–302. doi: 10.2353/ajpath.2006.060185 PMC1698845 17003486

[218] Lockwood CJ Murk WK Kayisli UA Buchwalder LF Huang SJ Arcuri F. Regulation of interleukin-6 expression in human decidual cells and its potential role in chorioamnionitis. Am J Pathol (2010) 177:1755–64. doi: 10.2353/ajpath.2010.090781 PMC2947272 20724602

[219] Guzeloglu-Kayisli O Kayisli UA Semerci N Basar M Buchwalder LF Buhimschi CS. Mechanisms of chorioamnionitis-associated preterm birth: interleukin-1β inhibits progesterone receptor expression in decidual cells. J Pathol (2015) 237:423–34. doi: 10.1002/path.4589 26175191

[220] Cha J Bartos A Egashira M Haraguchi H Saito-Fujita T Leishman E. Combinatory approaches prevent preterm birth profoundly exacerbated by gene-environment interactions. J Clin Invest (2013) 123:4063–75. doi: 10.1172/jci70098 PMC3754274 23979163

[221] Rinaldi SF Makieva S Saunders PT Rossi AG Norman JE. Immune cell and transcriptomic analysis of the human decidua in term and preterm parturition. Mhr Basic Sci Reprod Med (2017) 23:708–24. doi: 10.1093/molehr/gax038 PMC5909855 28962035

[222] Zhang G Feenstra B Bacelis J Liu X Muglia LM Juodakis J. Genetic associations with gestational length and spontaneous preterm birth. New Engl J Med (2017) 377:1156–67. doi: 10.1056/nejmoa1612665 PMC5561422 28877031

[223] Liu X Helenius D Skotte L Beaumont RN Wielscher M Geller F. Variants in the fetal genome near pro-inflammatory cytokine genes on 2q13 associate with gestational duration. Nat Commun (2019) 10:3927. doi: 10.1038/s41467-019-11881-8 31477735 PMC6718389

[224] Sakabe NJ Aneas I Knoblauch N Sobreira DR Clark N Paz C. Transcriptome and regulatory maps of decidua-derived stromal cells inform gene discovery in preterm birth. Sci Adv (2020) 6:eabc8696. doi: 10.1126/sciadv.abc8696 33268355 PMC7710387

[225] Louis DS Romero R Plazyo O Arenas-Hernandez M Panaitescu B Xu Y. Invariant NKT cell activation induces late preterm birth that is attenuated by rosiglitazone. J Immunol (2016) 196:1044–59. doi: 10.4049/jimmunol.1501962 PMC4724534 26740111

[226] Lockwood CJ Murk W Kayisli UA Buchwalder LF Huang S-T Funai EF. Progestin and thrombin regulate tissue factor expression in human term decidual cells. J Clin Endocrinol Metab (2009) 94:2164–70. doi: 10.1210/jc.2009-0065 PMC2690421 19276228

[227] O’Sullivan CJ Allen NM O’Loughlin AJ Friel AM Morrison JJ. Thrombin and PAR1-activating peptide: effects on human uterine contractility. vitro. Am J Obstet Gynecol (2004) 190:1098–105. doi: 10.1016/j.ajog.2003.09.050 15118649

[228] Mackenzie AP Schatz F Krikun G Funai EF Kadner S Lockwood CJ. Mechanisms of abruption-induced premature rupture of the fetal membranes: Thrombin enhanced decidual matrix metalloproteinase-3 (stromelysin-1) expression. Am J Obstet Gynecol (2004) 191:1996–2001. doi: 10.1016/j.ajog.2004.08.003 15592282

[229] Lockwood CJ Toti P Arcuri F Paidas M Buchwalder L Krikun G. Mechanisms of abruption-induced premature rupture of the fetal membranes. Am J Pathol (2005) 167:1443–9. doi: 10.1016/s0002-9440(10)61230-8 PMC1603775 16251427

[230] Shynlova O Lee Y-H Srikhajon K Lye SJ. Physiologic uterine inflammation and labor onset: Integration of endocrine and mechanical signals. Reprod Sci (2013) 20:154–67. doi: 10.1177/1933719112446084 22614625

[231] Sivarajasingam SP Imami N Johnson MR. Myometrial cytokines and their role in the onset of labour. J Endocrinol (2016) 231:R101–19. doi: 10.1530/joe-16-0157 27647860

[232] Mendelson CR Gao L Montalbano AP. Multifactorial regulation of myometrial contractility during pregnancy and parturition. Front Endocrinol (2019) 10:714. doi: 10.3389/fendo.2019.00714 PMC6823183 31708868

[233] Nadeem L Shynlova O Matysiak-Zablocki E Mesiano S Dong X Lye S. Molecular evidence of functional progesterone withdrawal in human myometrium. Nat Commun (2016) 7:11565. doi: 10.1038/ncomms11565 27220952 PMC4894948

[234] Mesiano S Chan E-C Fitter JT Kwek K Yeo G Smith R. Progesterone withdrawal and estrogen activation in human parturition are coordinated by progesterone receptor a expression in the myometrium. J Clin Endocrinol Metab (2002) 87:2924–30. doi: 10.1210/jcem.87.6.8609 12050275

[235] Pabona JMP Zhang D Ginsburg DS Simmen FA Simmen RCM. Prolonged pregnancy in women is associated with attenuated myometrial expression of progesterone receptor co-regulator krüppel-like factor 9. J Clin Endocrinol Metab (2015) 100:166–74. doi: 10.1210/jc.2014-2846 PMC4283014 25313913

[236] Chai SY Smith R Fitter JT Mitchell C Pan X Ilicic M. Increased progesterone receptor a expression in labouring human myometrium is associated with decreased promoter occupancy by the histone demethylase JARID1A. Mol Hum Reprod (2014) 20:442–53. doi: 10.1093/molehr/gau005 24442343

[237] Ke W Chen C Luo H Tang J Zhang Y Gao W. Histone deacetylase 1 regulates the expression of progesterone receptor a during human parturition by occupying the progesterone receptor a promoter. Reprod Sci (2016) 23:955–64. doi: 10.1177/1933719115625848 26758364

[238] Runnebaum B Zander J. Progesterone and 20α-dihydroprogesterone in human myometrium during pregnancy. Acta Endocrinol-cop (1971) 65:S5–S50. doi: 10.1530/acta.0.066s005 5279327

[239] Williams KC Renthal NE Condon JC Gerard RD Mendelson CR. MicroRNA-200a serves a key role in the decline of progesterone receptor function leading to term and preterm labor. Proc Natl Acad Sci (2012) 109:7529–34. doi: 10.1073/pnas.1200650109 PMC3358858 22529366

[240] Nadeem L Balendran R Dorogin A Mesiano S Shynlova O Lye SJ. Pro-inflammatory signals induce 20α-HSD expression in myometrial cells: A key mechanism for local progesterone withdrawal. J Cell Mol Med (2021) 25:6773–85. doi: 10.1111/jcmm.16681 PMC8278114 34114342

[241] Arrowsmith S Wray S. Oxytocin: its mechanism of action and receptor signalling in the myometrium. J Neuroendocrinol (2014) 26:356–69. doi: 10.1111/jne.12154 24888645

[242] Nadeem L Shynlova O Mesiano S Lye S. Progesterone *Via* its type-a receptor promotes myometrial gap junction coupling. Sci Rep-uk (2017) 7:13357. doi: 10.1038/s41598-017-13488-9 PMC5645358 29042599

[243] Yoshida M Takayanagi Y Ichino-Yamashita A Sato K Sugimoto Y Kimura T. Functional hierarchy of uterotonics required for successful parturition in mice. Endocrinology (2019) 160:2800–10. doi: 10.1210/en.2019-00499 PMC6887699 31517984

[244] Pique-Regi R Romero R Garcia-Flores V Peyvandipour A Tarca AL Pusod E. A single-cell atlas of the myometrium in human parturition. JCI Insight (2022) 7:e153921. doi: 10.1172/jci.insight.153921 35260533 PMC8983148

[245] Singh N Herbert B Sooranna GR Orsi NM Edey L Dasgupta T. Is myometrial inflammation a cause or a consequence of term human labour? J Endocrinol (2017) 235:69–83. doi: 10.1530/joe-17-0318 28765265

[246] Shynlova O Tsui P Dorogin A Lye SJ. Monocyte chemoattractant protein-1 (CCL-2) integrates mechanical and endocrine signals that mediate term and preterm labor. J Immunol (2008) 181:1470–9. doi: 10.4049/jimmunol.181.2.1470 18606702

[247] Osman I Young A Ledingham MA Thomson AJ Jordan F Greer IA. Leukocyte density and pro-inflammatory cytokine expression in human fetal membranes, decidua, cervix and myometrium before and during labour at term. Mhr Basic Sci Reprod Med (2003) 9:41–5. doi: 10.1093/molehr/gag001 12529419

[248] Ledingham MA Thomson AJ Jordan F Young A Crawford M Norman JE. Cell adhesion molecule expression in the cervix and myometrium during pregnancy and parturition. Obstetrics Gynecol (2001) 97:235–42. doi: 10.1097/00006250-200102000-00014 11165588

[249] Thomson AJ Telfer JF Young A Campbell S Stewart CJR Cameron IT. Leukocytes infiltrate the myometrium during human parturition: further evidence that labour is an inflammatory process. Hum Reprod (1999) 14:229–36. doi: 10.1093/humrep/15.1.229 10374126

[250] Allport VC Pieber D Slater DM Newton R White JO Bennett PR. Human labour is associated with nuclear factor-kappaB activity which mediates cyclo-oxygenase-2 expression and is involved with the “functional progesterone withdrawal.” Mol Hum Reprod (2001) 7:581–6. doi: 10.1093/molehr/7.6.581 11385114

[251] Romero R Espinoza J Gonçalves L Kusanovic J Friel L Hassan S. The role of inflammation and infection in preterm birth. Semin Reprod Med (2007) 25:021–39. doi: 10.1055/s-2006-956773 PMC8324073 17205421

[252] Lim R Barker G Lappas M. PARK7 regulates inflammation-induced pro-labour mediators in myometrial and amnion cells. Reproduction (2018) 155:207–18. doi: 10.1530/rep-17-0604 29358306

[253] Kadam L Gomez-Lopez N Mial TN Kohan-Ghadr H-R Drewlo S. Rosiglitazone regulates TLR4 and rescues HO-1 and NRF2 expression in myometrial and decidual macrophages in inflammation-induced preterm birth. Reprod Sci (2017) 24:1590–9. doi: 10.1177/1933719117697128 PMC6728562 28322133

[254] Hutchinson JL Rajagopal SP Yuan M Norman JE. Lipopolysaccharide promotes contraction of uterine myocytes *via* activation of Rho/ROCK signaling pathways. FASEB J Off Publ Fed Am Soc Exp Biol (2013) 28:94–105. doi: 10.1096/fj.13-237040 24076962

[255] Lim R Lappas M. GIT2 deficiency attenuates inflammation induced expression of pro-labor mediators in human amnion and myometrial cells. Biol Reprod (2019) 100:1617–29. doi: 10.1093/biolre/ioz041 30915469

[256] Goradia P Lim R Lappas M. DREAM is involved in the genesis of inflammation-induced prolabour mediators in human myometrial and amnion cells. BioMed Res Int (2018) 2018:8237087. doi: 10.1155/2018/8237087 29682558 PMC5842746

[257] Nadeau-Vallee M Boudreault A Leimert K Hou X Obari D Madaan A. Uterotonic neuromedin U receptor 2 and its ligands are upregulated by inflammation in mice and humans, and elicit preterm birth. Biol Reprod (2016) 95:72–2. doi: 10.1095/biolreprod.116.140905 PMC5394981 27512149

[258] Migale R MacIntyre DA Cacciatore S Lee YS Hagberg H Herbert BR. Modeling hormonal and inflammatory contributions to preterm and term labor using uterine temporal transcriptomics. BMC Med (2016) 14:86. doi: 10.1186/s12916-016-0632-4 27291689 PMC4904357

[259] Keski-Nisula LT Aalto M-L Kirkinen PP Kosma V-M Heinonen ST. Myometrial inflammation in human delivery and its association with labor and infection. Am J Clin Pathol (2003) 120:217–24. doi: 10.1309/kc6kdtx98lfyb3j7 12931552

[260] Tattersall M Engineer N Khanjani S Sooranna SR Roberts VH Grigsby PL. Pro-labour myometrial gene expression: are preterm labour and term labour the same? Reproduction (2008) 135:569–79. doi: 10.1530/rep-07-0461 18367515

[261] Weiner CP Mason CW Dong Y Buhimschi IA Swaan PW Buhimschi CS. Human effector/initiator gene sets that regulate myometrial contractility during term and preterm labor. Am J Obstet Gynecol (2010) 202:474.e1–20. doi: 10.1016/j.ajog.2010.02.034 PMC2867841 20452493

[262] Renthal NE Chen C-C Williams KC Gerard RD Prange-Kiel J Mendelson CR. miR-200 family and targets, ZEB1 and ZEB2, modulate uterine quiescence and contractility during pregnancy and labor. Proc Natl Acad Sci (2010) 107:20828–33. doi: 10.1073/pnas.1008301107 PMC2996411 21079000

[263] Tang Y Ji H Liu H Gu W Li X Peng T. Identification and functional analysis of microRNA in myometrium tissue from spontaneous preterm labor. Int J Clin Exp Patho (2015) 8:12811–9. PMC4680416 26722471

[264] Gao L Wang G Liu W-N Kinser H Franco HL Mendelson CR. Reciprocal feedback between miR-181a and E2/ERα in myometrium enhances inflammation leading to labor. J Clin Endocrinol Metab (2016) 101:3646–56. doi: 10.1210/jc.2016-2078 PMC5052345 27459534

[265] Yao Y Robinson AM Zucchi FC Robbins JC Babenko O Kovalchuk O. Ancestral exposure to stress epigenetically programs preterm birth risk and adverse maternal and newborn outcomes. BMC Med (2014) 12:121. doi: 10.1186/s12916-014-0121-6 25286408 PMC4244860

[266] Gray C McCowan LM Patel R Taylor RS Vickers MH. Maternal plasma miRNAs as biomarkers during mid-pregnancy to predict later spontaneous preterm birth: a pilot study. Sci Rep-uk (2017) 7:815. doi: 10.1038/s41598-017-00713-8 PMC5429750 28400603

[267] Winger EE Reed JL Ji X Gomez-Lopez N Pacora P Romero R. MicroRNAs isolated from peripheral blood in the first trimester predict spontaneous preterm birth. PloS One (2020) 15:e0236805. doi: 10.1371/journal.pone.0236805 32790689 PMC7425910

[268] Stock S Norman J. Preterm and term labour in multiple pregnancies. Semin Fetal Neonatal Med (2010) 15:336–41. doi: 10.1016/j.siny.2010.06.006 20643592

[269] Bacelis J Juodakis J Waldorf KMA Sengpiel V Muglia LJ Zhang G. Uterine distention as a factor in birth timing: retrospective nationwide cohort study in Sweden. BMJ Open (2018) 8:e022929. doi: 10.1136/bmjopen-2018-022929 PMC6252709 30385442

[270] Ou C-W Orsino A Lye SJ. Expression of connexin-43 and connexin-26 in the rat myometrium during pregnancy and labor is differentially regulated by mechanical and hormonal signals. Endocrinology (1997) 138:5398–407. doi: 10.1210/endo.138.12.5624 9389525

[271] Terzidou V Sooranna SR Kim LU Thornton S Bennett PR Johnson MR. Mechanical stretch up-regulates the human oxytocin receptor in primary human uterine myocytes. J Clin Endocrinol Metab (2005) 90:237–46. doi: 10.1210/jc.2004-0277 15494465

[272] Sooranna SR Lee Y Kim LU Mohan AR Bennett PR Johnson MR. Mechanical stretch activates type 2 cyclooxygenase *via* activator protein-1 transcription factor in human myometrial cells. Mhr Basic Sci Reprod Med (2004) 10:109–13. doi: 10.1093/molehr/gah021 14742695

[273] Wathes DC Porter DG. Effect of uterine distension and oestrogen treatment on gap junction formation in the myometrium of the rat. Reproduction (1982) 65:497–505. doi: 10.1530/jrf.0.0650497 7097654

[274] Waldorf KMA Singh N Mohan AR Young RC Ngo L Das A. Uterine overdistention induces preterm labor mediated by inflammation: observations in pregnant women and nonhuman primates. Am J Obstet Gynecol (2015) 213:830.e1–830.e19. doi: 10.1016/j.ajog.2015.08.028 26284599 PMC4679421

[275] Elovitz MA Baron J Phillippe M. The role of thrombin in preterm parturition. Am J Obstet Gynecol (2001) 185:1059–63. doi: 10.1067/mob.2001.117638 11717633

[276] Lockwood CJ Kayisli UA Stocco C Murk W Vatandaslar E Buchwalder LF. Abruption-induced preterm delivery is associated with thrombin-mediated functional progesterone withdrawal in decidual cells. Am J Pathol (2012) 181:2138–48. doi: 10.1016/j.ajpath.2012.08.036 PMC3509762 23058370

[277] Nishimura F Mogami H Moriuchi K Chigusa Y Mandai M Kondoh E. Mechanisms of thrombin-induced myometrial contractions: Potential targets of progesterone. PloS One (2020) 15:e0231944. doi: 10.1371/journal.pone.0231944 32365105 PMC7197857

[278] Xu R Meng X Pang Y An H Wang B Zhang L. Associations of maternal exposure to 41 metals/metalloids during early pregnancy with the risk of spontaneous preterm birth: Does oxidative stress or DNA methylation play a crucial role? Environ Int (2022) 158:106966. doi: 10.1016/j.envint.2021.106966 34735952

[279] Shepherd MC Radnaa E Tantengco OA Kechichian T Urrabaz-Garza R Kammala AK. Extracellular vesicles from maternal uterine cells exposed to risk factors cause fetal inflammatory response. Cell Commun Signal Ccs (2021) 19:100. doi: 10.1186/s12964-021-00782-3 34620169 PMC8499538

[280] Nott JP Bonney EA Pickering JD Simpson NAB. The structure and function of the cervix during pregnancy. Transl Res Anat (2016) 2:1–7. doi: 10.1016/j.tria.2016.02.001

[281] Yellon SM. Contributions to the dynamics of cervix remodeling prior to term and preterm birth. Biol Reprod (2016) 96:13–23. doi: 10.1095/biolreprod.116.142844 PMC5803764 28395330

[282] Yellon SM. Immunobiology of cervix ripening. Front Immunol (2020) 10:3156. doi: 10.3389/fimmu.2019.03156 32038651 PMC6993120

[283] Word1 R Li1 XH Hnat1 M Carrick K. Dynamics of cervical remodeling during pregnancy and parturition: Mechanisms and current concepts. Semin Reprod Med (2007) 25:069–79. doi: 10.1055/s-2006-956777 17205425

[284] Vink J Yu V Dahal S Lohner J Stern-Asher C Mourad M. Extracellular matrix rigidity modulates human cervical smooth muscle contractility–new insights into premature cervical failure and spontaneous preterm birth. Reprod Sci (2021) 28:237–51. doi: 10.1007/s43032-020-00268-6 PMC9344974 32700284

[285] Vink JY Qin S Brock CO Zork NM Feltovich HM Chen X. A new paradigm for the role of smooth muscle cells in the human cervix. Am J Obstet Gynecol (2016) 215:478.e1–478.e11. doi: 10.1016/j.ajog.2016.04.053 27166013

[286] Tantengco OAG Menon R. Contractile function of the cervix plays a role in normal and pathological pregnancy and parturition. Med Hypotheses (2020) 145:110336. doi: 10.1016/j.mehy.2020.110336 33049595 PMC7899741

[287] Timmons B Akins M Mahendroo M. Cervical remodeling during pregnancy and parturition. Trends Endocrinol Metab (2010) 21:353–61. doi: 10.1016/j.tem.2010.01.011 PMC2880223 20172738

[288] Timmons BC Mitchell SM Gilpin C Mahendroo MS. Dynamic changes in the cervical epithelial tight junction complex and differentiation occur during cervical ripening and parturition. Endocrinology (2007) 148:1278–87. doi: 10.1210/en.2006-0851 17138657

[289] Hein M Valore EV Helmig RB Uldbjerg N Ganz T. Antimicrobial factors in the cervical mucus plug. Am J Obstet Gynecol (2002) 187:137–44. doi: 10.1067/mob.2002.123034 12114901

[290] Hansen LK Becher N Bastholm S Glavind J Ramsing M Kim CJ. The cervical mucus plug inhibits, but does not block, the passage of ascending bacteria from the vagina during pregnancy. Acta Obstet Gyn Scan (2014) 93:102–8. doi: 10.1111/aogs.12296 PMC5987199 24266587

[291] Tantengco OAG Menon R. Breaking down the barrier: The role of cervical infection and inflammation in preterm birth. Front Global Women’s Heal (2022) 2:777643. doi: 10.3389/fgwh.2021.777643 PMC8803751 35118439

[292] Nallasamy S Mahendroo M. Distinct roles of cervical epithelia and stroma in pregnancy and parturition. Semin Reprod Med (2017) 35:190–200. doi: 10.1055/s-0037-1599091 28278536

[293] Hezelgrave NL Seed PT Chin-Smith EC Ridout AE Shennan AH Tribe RM. Cervicovaginal natural antimicrobial expression in pregnancy and association with spontaneous preterm birth. Sci Rep-uk (2020) 10:12018. doi: 10.1038/s41598-020-68329-z PMC7374562 32694552

[294] Suff N Karda R Diaz JA Ng J Baruteau J Perocheau D. Cervical gene delivery of the antimicrobial peptide, human β-defensin (HBD)-3, in a mouse model of ascending infection-related preterm birth. Front Immunol (2020) 11:106. doi: 10.3389/fimmu.2020.00106 32117260 PMC7026235

[295] Tantengco OAG Kechichian T Vincent KL Pyles RB Medina PMB Menon R. Inflammatory response elicited by ureaplasma parvum colonization in human cervical epithelial, stromal, and immune cells. Reprod Camb Engl (2021) 163:1–10. doi: 10.1530/rep-21-0308 PMC8669769 34780348

[296] Dubicke A Fransson E Centini G Andersson E Byström B Malmström A. Pro-inflammatory and anti-inflammatory cytokines in human preterm and term cervical ripening. J Reprod Immunol (2010) 84:176–85. doi: 10.1016/j.jri.2009.12.004 20096464

[297] Mattsby-Baltzer I Platz-Christensen JJ Hosseini N Rosén P. IL-1beta, IL-6, TNFalpha, fetal fibronectin, and endotoxin in the lower genital tract of pregnant women with bacterial vaginosis. Acta Obstet Gyn Scan (1998) 77:701–6. 9740515

[298] Sawada M Otsuki K Mitsukawa K Yakuwa K Nagatsuka M Okai T. Cervical inflammatory cytokines and other markers in the cervical mucus of pregnant women with lower genital tract infection. Int J Gynecol Obstet (2006) 92:117–21. doi: 10.1016/j.ijgo.2005.10.004 16307744

[299] Simhan HN Caritis SN Hillier SL Krohn MA. Cervical anti-inflammatory cytokine concentrations among first-trimester pregnant smokers. Am J Obstet Gynecol (2005) 193:1999–2003. doi: 10.1016/j.ajog.2005.04.054 16325603

[300] Simhan HN Ryckman KK Williams SM Krohn MA. Genetic regulation of cervical antiinflammatory cytokine concentrations during pregnancy. Am J Obstet Gynecol (2008) 199:163.e1–163.e11. doi: 10.1016/j.ajog.2008.02.033 18674658

[301] Sennström MB Ekman G Westergren-Thorsson G Malmström A Byström B Endrésen U. Human cervical ripening, an inflammatory process mediated by cytokines. Mhr Basic Sci Reprod Med (2000) 6:375–81. doi: 10.1093/molehr/6.4.375 10729321

[302] Read CP Word RA Ruscheinsky MA Timmons BC Mahendroo MS. Cervical remodeling during pregnancy and parturition: molecular characterization of the softening phase in mice. Reproduction (2007) 134:327–40. doi: 10.1530/rep-07-0032 17660242

[303] Payne KJ Clyde LA Weldon AJ Milford T-A Yellon SM. Residency and activation of myeloid cells during remodeling of the prepartum murine cervix. Biol Reprod (2012) 87:106. doi: 10.1095/biolreprod.112.101840 22914314 PMC3509777

[304] Dubicke A Ekman-Ordeberg G Mazurek P Miller L Yellon SM. Density of stromal cells and macrophages associated with collagen remodeling in the human cervix in preterm and term birth. Reprod Sci (2016) 23:595–603. doi: 10.1177/1933719115616497 26608218 PMC5933160

[305] Stjernholm-Vladic Y Stygar D Mansson C Masironi B Akerberg S Wang H. Factors involved in the inflammatory events of cervical ripening in humans. Reprod Biol Endocrin (2004) 2:74. doi: 10.1186/1477-7827-2-74 PMC534613 15500686

[306] Roos N Blesson CS Stephansson O Masironi B Stjernholm YV Ekman-Ordeberg G. The expression of prostaglandin receptors EP3 and EP4 in human cervix in post-term pregnancy differs between failed and successful labor induction. Acta Obstet Gyn Scan (2014) 93:159–67. doi: 10.1111/aogs.12300 24180609

[307] Heuerman AC Hollinger TT Menon R Mesiano S Yellon SM. Cervix stromal cells and the progesterone receptor a isoform mediate effects of progesterone for prepartum remodeling. Reprod Sci (2019) 26:690–6. doi: 10.1177/1933719118820462 PMC6728580 30654718

[308] Ackerman WE Summerfield TL Mesiano S Schatz F Lockwood CJ Kniss DA. Agonist-dependent downregulation of progesterone receptors in human cervical stromal fibroblasts. Reprod Sci (2016) 23:112–23. doi: 10.1177/1933719115597787 PMC5933193 26243545

[309] Ramos JG Varayoud J Bosquiazzo VL Luque EH Muñoz-de-Toro M. Cellular turnover in the rat uterine cervix and its relationship to estrogen and progesterone receptor dynamics. Biol Reprod (2002) 67:735–42. doi: 10.1095/biolreprod.101.002402 12193379

[310] Heng YJ Liong S Permezel M Rice GE Quinzio MKWD Georgiou HM. Human cervicovaginal fluid biomarkers to predict term and preterm labor. Front Physiol (2015) 6:151. doi: 10.3389/fphys.2015.00151 26029118 PMC4429550

[311] Heng YJ Quinzio MKWD Permezel M Rice GE Georgiou HM. Temporal expression of antioxidants in human cervicovaginal fluid associated with spontaneous labor. Antioxid Redox Sign (2010) 13:951–7. doi: 10.1089/ars.2010.3122 20446766

[312] Sahlin L Wang H Stjernholm Y Lundberg M Ekman G Holmgren A. The expression of glutaredoxin is increased in the human cervix in term pregnancy and immediately post-partum, particularly after prostaglandin-induced delivery. Mhr Basic Sci Reprod Med (2000) 6:1147–53. doi: 10.1093/molehr/6.12.1147 11101698

[313] Tantengco OAG Vink J Medina PMB Menon R. Oxidative stress promotes cellular damages in the cervix: implications for normal and pathologic cervical function in human pregnancy. Biol Reprod (2021) 105:204–16. doi: 10.1093/biolre/ioab058 PMC8256103 33760067

[314] AD ALLAIRE D’andrea N Truong P Mj M Lessey BA. Cervical stroma apoptosis in pregnancy. Obstetrics Gynecol (2001) 97:399–403. doi: 10.1097/00006250-200103000-00015 11239645

[315] Tantengco OAG Richardson LS Medina PMB Han A Menon R. Organ-on-chip of the cervical epithelial layer: A platform to study normal and pathological cellular remodeling of the cervix. FASEB J (2021) 35:e21463. doi: 10.1096/fj.202002590rrr 33689188 PMC8193817

[316] Gordon J Mowa CN. Mechanobiology of mice cervix: expression profile of mechano-related molecules during pregnancy. Cell Tissue Res (2019) 376:443–56. doi: 10.1007/s00441-018-02983-8 30671632

[317] Hassan SS Romero R Tarca AL Nhan-Chang C-L Vaisbuch E Erez O. The transcriptome of cervical ripening in human pregnancy before the onset of labor at term: Identification of novel molecular functions involved in this process. J Maternal-fetal Neonatal Med (2009) 22:1183–93. doi: 10.3109/14767050903353216 PMC7062290 19883264

[318] Tantengco OAG Richardson LS Menon R. Effects of a gestational level of estradiol on cellular transition, migration, and inflammation in cervical epithelial and stromal cells. Am J Reprod Immunol (2021) 85:e13370. doi: 10.1111/aji.13370 33152143 PMC9371442

[319] Tantengco OAG Richardson LS Vink J Kechichian T Medina PMB Pyles RB. Progesterone alters human cervical epithelial and stromal cell transition and migration: Implications in cervical remodeling during pregnancy and parturition. Mol Cell Endocrinol (2021) 529:111276. doi: 10.1016/j.mce.2021.111276 33823217 PMC8491272

[320] Kishore AH Li X-H Word RA. Hypoxia and PGE(2) regulate MiTF-CX during cervical ripening. Mol Endocrinol Baltim Md (2012) 26:2031–45. doi: 10.1210/me.2012-1100 PMC3517718 23144021

[321] Word RA Stull JT Casey ML Kamm KE. Contractile elements and myosin light chain phosphorylation in myometrial tissue from nonpregnant and pregnant women. J Clin Invest (1993) 92:29–37. doi: 10.1172/jci116564 8392087 PMC293522

[322] Nallasamy S Yoshida K Akins M Myers K Iozzo R Mahendroo M. Steroid hormones are key modulators of tissue mechanical function *via* regulation of collagen and elastic fibers. Endocrinology (2017) 158:950–62. doi: 10.1210/en.2016-1930 PMC5460796 28204185

[323] Mahendroo M. Cervical remodeling in term and preterm birth: insights from an animal model. Reproduction (2012) 143:429–38. doi: 10.1530/rep-11-0466 22344465

[324] Nallasamy S Palacios HH Setlem R Caraballo MC Li K Cao E. Transcriptome and proteome dynamics of cervical remodeling in the mouse during pregnancy. Biol Reprod (2021) 105:1257–71. doi: 10.1093/biolre/ioab144 PMC8599062 34309663

[325] Cooley A Madhukaran S Stroebele E Caraballo MC Wang L Hon GC. Dynamic states of cervical epithelia during pregnancy and epithelial barrier disruption. Biorxiv (2022) 2022:7.26.501609. doi: 10.1101/2022.07.26.501609 PMC9883190 36718364

[326] Diez S Renner M Bahlinger V Hartmann A Besendörfer M Müller H. Increased expression of OLFM4 and lysozyme during necrotizing enterocolitis in neonates: an observational research study. BMC Pediatr (2022) 22:192. doi: 10.1186/s12887-022-03260-y 35410162 PMC8996401

[327] Tiensuu H Haapalainen AM Tissarinen P Pasanen A Määttä TA Huusko JM. Human placental proteomics and exon variant studies link AAT/SERPINA1 with spontaneous preterm birth. BMC Med (2022) 20:141. doi: 10.1186/s12916-022-02339-8 35477570 PMC9047282

[328] Akgul Y Word RA Ensign LM Yamaguchi Y Lydon J Hanes J. Hyaluronan in cervical epithelia protects against infection-mediated preterm birth. J Clin Invest (2014) 124:5481–9. doi: 10.1172/jci78765 PMC4348952 25384213

[329] Menon R Bonney EA Condon J Mesiano S Taylor RN. Novel concepts on pregnancy clocks and alarms: redundancy and synergy in human parturition. Hum Reprod Update (2016) 22:535–60. doi: 10.1093/humupd/dmw022 PMC5001499 27363410

[330] Andersson S Minjarez D Yost NP Word RA. Estrogen and progesterone metabolism in the cervix during pregnancy and parturition. J Clin Endocrinol Metab (2008) 93:2366–74. doi: 10.1210/jc.2007-2813 PMC2435631 18364378

[331] Chwalisz K Garfield RE. Role of nitric oxide in the uterus and cervix: Implications for the management of labor. J Perinat Med (1998) 26:448–57. doi: 10.1515/jpme.1998.26.6.448 10224601

[332] Kelly AJ Munson C Minden L. Nitric oxide donors for cervical ripening and induction of labour. Cochrane Db Syst Rev (2011) 16, CD006901. doi: 10.1002/14651858.cd006901.pub2 21678363

[333] Ekerhovd E Brännström M Weijdegård B Norström A. Nitric oxide synthases in the human cervix at term pregnancy and effects of nitric oxide on cervical smooth muscle contractility. Am J Obstet Gynecol (2000) 183:610–6. doi: 10.1067/mob.2000.105901 10992181

[334] Vink J Feltovich H. Cervical etiology of spontaneous preterm birth. Semin Fetal Neonatal Med (2016) 21:106–12. doi: 10.1016/j.siny.2015.12.009 PMC4798922 26776146

[335] Vink J Myers K. Cervical alterations in pregnancy. Best Pract Res Cl Ob (2018) 52:88–102. doi: 10.1016/j.bpobgyn.2018.03.007 PMC6282836 30314740

[336] Volozonoka L Rots D Kempa I Kornete A Rezeberga D Gailite L. Genetic landscape of preterm birth due to cervical insufficiency: Comprehensive gene analysis and patient next-generation sequencing data interpretation. PloS One (2020) 15:e0230771. doi: 10.1371/journal.pone.0230771 32214361 PMC7098624

[337] Wong LF Wilkes J Korgenski K Varner MW Manuck TA. Intrapartum cervical laceration and subsequent pregnancy outcomes. Am J Perinatol Rep (2016) 06:e318–23. doi: 10.1055/s-0036-1592198 PMC5017884 27621953

[338] Hamou B Sheiner E Coreanu T Walfisch A Silberstein T. Intrapartum cervical lacerations and their impact on future pregnancy outcome. J Maternal-fetal Neonatal Med (2018) 33:1–5. doi: 10.1080/14767058.2018.1505852 30189764

[339] Wittmaack A Dudley D Boyle A. Maternal history of cervical surgery and preterm delivery: A retrospective cohort study. J Women’s Heal (2019) 28:1538–42. doi: 10.1089/jwh.2018.7457 31730425

[340] Kindinger LM Kyrgiou M MacIntyre DA Cacciatore S Yulia A Cook J. Preterm birth prevention post-conization: A model of cervical length screening with targeted cerclage. PloS One (2016) 11:e0163793. doi: 10.1371/journal.pone.0163793 27812088 PMC5094773

[341] Holt R Timmons BC Akgul Y Akins ML Mahendroo M. The molecular mechanisms of cervical ripening differ between term and preterm birth. Endocrinology (2011) 152:1036–46. doi: 10.1210/en.2010-1105 PMC3040055 21209014

[342] Gonzalez JM Dong Z Romero R Girardi G. Cervical Remodeling/Ripening at term and preterm delivery: The same mechanism initiated by different mediators and different effector cells. PloS One (2011) 6:e26877. doi: 10.1371/journal.pone.0026877 22073213 PMC3206857

[343] Donders GG Calsteren CV Bellen G Reybrouck R den BTV Riphagen I. Association between abnormal vaginal flora and cervical length as risk factors for preterm birth. Ultrasound Obstetrics Gynecol (2010) 1–10. doi: 10.1002/uog.7568 20104531

[344] Gerson KD McCarthy C Elovitz MA Ravel J Sammel MD Burris HH. Cervicovaginal microbial communities deficient in lactobacillus species are associated with second trimester short cervix. Am J Obstet Gynecol (2020) 222:491.e1–8. doi: 10.1016/j.ajog.2019.11.1283 PMC7196011 31816307

[345] Paola MD Seravalli V Paccosi S Linari C Parenti A Filippo CD. Identification of vaginal microbial communities associated with extreme cervical shortening in pregnant women. J Clin Med (2020) 9:3621. doi: 10.3390/jcm9113621 PMC7698214 33182750

[346] Slager J Lynne S. Assessment of cervical length and the relationship between short cervix and preterm birth. J Midwifery Women S Heal (2012) 57:4–11. doi: 10.1111/j.1542-2011.2012.00209.x 22776243

[347] Arabin B Alfirevic Z. Cervical pessaries for prevention of spontaneous preterm birth: past, present and future. Ultrasound Obst Gyn (2013) 42:390–9. doi: 10.1002/uog.12540 PMC4282542 23775862

[348] Vink J Mourad M. The pathophysiology of human premature cervical remodeling resulting in spontaneous preterm birth: Where are we now? Semin Perinatol (2017) 41:427–37. doi: 10.1053/j.semperi.2017.07.014 PMC6007872 28826790

[349] Han Y Li M Ma H Yang H. Cervical insufficiency: a noteworthy disease with controversies. J Perinat Med (2020) 48:648–55. doi: 10.1515/jpm-2020-0255 32692707

[350] Nold C Anton L Brown A Elovitz M. Inflammation promotes a cytokine response and disrupts the cervical epithelial barrier: a possible mechanism of premature cervical remodeling and preterm birth. Am J Obstet Gynecol (2012) 206:208. doi: 10.1016/j.ajog.2011.12.036 22285171

[351] Raiche E Ouellet A Berthiaume M Rousseau αÉ Pasquier J-C. Short and inflamed cervix predicts spontaneous preterm birth (COLIBRI study). J Maternal-fetal Neonatal Med (2013) 27:1015–9. doi: 10.3109/14767058.2013.847917 24228627

[352] Shahin AY Hassanin IMA Ismail AM Kruessel JS Hirchenhain J. Effect of oral n-acetyl cysteine on recurrent preterm labor following treatment for bacterial vaginosis. Int J Gynecol Obstet (2009) 104:44–8. doi: 10.1016/j.ijgo.2008.08.026 18851855

[353] Tantengco OAG Radnaa E Shahin H Kechichian T Menon R. Cross talk: trafficking and functional impact of maternal exosomes at the feto-maternal interface under normal and pathologic states. Biol Reprod (2021) 105:1562–76. doi: 10.1093/biolre/ioab181 34554204

[354] Ðogić LM Lučić N Mićić D Omeragić F Hodžić E Fazlagić S. Correlation between cervical infection and preterm labor. Medicinski Glasnik Off Publ Med Assoc Zenica-doboj Canton Bosnia Herzegovina (2017) 14:91–7. doi: 10.17392/886-16 28165444

[355] McGee D Smith A Poncil S Patterson A Bernstein AI Racicot K. Cervical HSV-2 infection causes cervical remodeling and increases risk for ascending infection and preterm birth. PloS One (2017) 12:e0188645. doi: 10.1371/journal.pone.0188645 29190738 PMC5708831

[356] Racicot K Cardenas I Wünsche V Aldo P Guller S Means RE. Viral infection of the pregnant cervix predisposes to ascending bacterial infection. J Immunol (2013) 191:934–41. doi: 10.4049/jimmunol.1300661 PMC4153356 23752614

[357] Vornhagen J Quach P Santana-Ufret V Alishetti V Brokaw A Armistead B. Human cervical mucus plugs exhibit insufficiencies in antimicrobial activity towards group b streptococcus. J Infect Dis (2018) 217:1626–36. doi: 10.1093/infdis/jiy076 PMC5913629 29425317

[358] Anton L Sierra L-J DeVine A Barila G Heiser L Brown AG. Common cervicovaginal microbial supernatants alter cervical epithelial function: Mechanisms by which lactobacillus crispatus contributes to cervical health. Front Microbiol (2018) 9:2181. doi: 10.3389/fmicb.2018.02181 30349508 PMC6186799

[359] Pavlidis I Spiller OB Demarco GS MacPherson H Howie SEM Norman JE. Cervical epithelial damage promotes ureaplasma parvum ascending infection, intrauterine inflammation and preterm birth induction in mice. Nat Commun (2020) 11:199. doi: 10.1038/s41467-019-14089-y 31924800 PMC6954262

[360] Bevis KS Biggio JR. Cervical conization and the risk of preterm delivery. Am J Obstet Gynecol (2011) 205:19–27. doi: 10.1016/j.ajog.2011.01.003 21345402

[361] Anton L DeVine A Sierra L-J Brown AG Elovitz MA. miR-143 and miR-145 disrupt the cervical epithelial barrier through dysregulation of cell adhesion, apoptosis and proliferation. Sci Rep-uk (2017) 7:3020. doi: 10.1038/s41598-017-03217-7 PMC5465080 28596604

[362] Russell RS Igietseme JU Eko FO. Exosomes: A possible mechanism for the development of chlamydia-induced upper genital tract pathology. J Immunol (2016) 1 Supplement:66:4.

[363] Nishiumi F Ogawa M Nakura Y Hamada Y Nakayama M Mitobe J. Intracellular fate of ureaplasma parvum entrapped by host cellular autophagy. Microbiologyopen (2017) 6:e00441. doi: 10.1002/mbo3.441 PMC5458467 28088841

[364] Care A Nevitt SJ Medley N Donegan S Good L Hampson L. Interventions to prevent spontaneous preterm birth in women with singleton pregnancy who are at high risk: systematic review and network meta-analysis. Bmj (2022) 376:e064547. doi: 10.1136/bmj-2021-064547 35168930 PMC8845039

[365] Bulletins—Obstetrics AC of O and GC on P. Prediction and prevention of spontaneous preterm birth: ACOG practice bulletin, number 234. Obstetrics Gynecol (2021) 138:e65–90. doi: 10.1097/aog.0000000000004479 34293771

[366] Simhan HN Caritis SN. Prevention of preterm delivery. New Engl J Med (2007) 357:477–87. doi: 10.1056/nejmra050435 17671256

[367] Nielsen BW Bonney EA Pearce BD Donahue LR Sarkar IN. (PREBIC) PBIC. a cross-species analysis of animal models for the investigation of preterm birth mechanisms. Reprod Sci (2016) 23:482–91. doi: 10.1177/1933719115604729 PMC5933186 26377998

[368] Ratajczak CK Fay JC Muglia LJ. Preventing preterm birth: the past limitations and new potential of animal models. Dis Model Mech (2010) 3:407–14. doi: 10.1242/dmm.001701 20610693

[369] Manuel CR Ashby CR Reznik SE. Discrepancies in animal models of preterm birth. Curr Pharm Design (2018) 23:6142–8. doi: 10.2174/1381612823666171012101114 29022513

[370] Leung CM de HP Ronaldson-Bouchard K Kim G-A Ko J HS R. A guide to the organ-on-a-chip. Nat Rev Methods Primers (2022) 2:33. doi: 10.1038/s43586-022-00118-6

[371] Novak R Ingram M Marquez S Das D Delahanty A Herland A. Robotic fluidic coupling and interrogation of multiple vascularized organ chips. Nat BioMed Eng (2020) 4:407–20. doi: 10.1038/s41551-019-0497-x PMC8057865 31988458

[372] Ma C Peng Y Li H Chen W. Organ-on-a-Chip: A new paradigm for drug development. Trends Pharmacol Sci (2020) 42:119–33. doi: 10.1016/j.tips.2020.11.009 PMC7990030 33341248

[373] Richardson L Kim S Menon R Han A. Organ-On-Chip technology: The future of feto-maternal interface research? Front Physiol (2020) 11:715. doi: 10.3389/fphys.2020.00715 32695021 PMC7338764

